# Parallel quorum-sensing system in *Vibrio cholerae* prevents signal interference inside the host

**DOI:** 10.1371/journal.ppat.1008313

**Published:** 2020-02-14

**Authors:** Samit Watve, Kelsey Barrasso, Sarah A. Jung, Kristen J. Davis, Lisa A. Hawver, Atul Khataokar, Ryan G. Palaganas, Matthew B. Neiditch, Lark J. Perez, Wai-Leung Ng

**Affiliations:** 1 Department of Molecular Biology and Microbiology, Tufts University School of Medicine, Boston, Massachusetts, United States of America; 2 Program in Molecular Microbiology, Tufts University, Graduate School of Biomedical Sciences, Boston, Massachusetts, United States of America; 3 Department of Microbiology, Biochemistry, and Molecular Genetics, New Jersey Medical School, Rutgers, The State University of New Jersey, Newark, New Jersey, United States of America; 4 Department of Chemistry & Biochemistry, Rowan University, Glassboro, New Jersey, United States of America; UC Santa Cruz, UNITED STATES

## Abstract

Many bacteria use quorum sensing (QS) to regulate virulence factor production in response to changes in population density. QS is mediated through the production, secretion, and detection of signaling molecules called autoinducers (AIs) to modulate population-wide behavioral changes. Four histidine kinases, LuxPQ, CqsS, CqsR and VpsS, have been identified in *Vibrio cholerae* as QS receptors to activate virulence gene expression at low cell density. Detection of AIs by these receptors leads to virulence gene repression at high cell density. The redundancy among these receptors is puzzling since any one of the four receptors is sufficient to support colonization of *V*. *cholerae* in the host small intestine. It is believed that one of the functions of such circuit architecture is to prevent interference on any single QS receptor. However, it is unclear what natural molecules can interfere *V*. *cholerae* QS and in what environment interference is detrimental. We show here mutants expressing only CqsR without the other three QS receptors are defective in colonizing the host large intestine. We identified ethanolamine, a common intestinal metabolite that can function as a chemical source of QS interference. Ethanolamine specifically interacts with the ligand-binding CACHE domain of CqsR and induces a premature QS response in *V*. *cholerae* mutants expressing only CqsR without the other three QS receptors. The effect of ethanolamine on QS gene expression and host colonization is abolished by mutations that disrupt CqsR signal sensing. *V*. *cholerae* defective in producing ethanolamine is still proficient in QS, therefore, ethanolamine functions only as an external cue for CqsR. Our findings suggest the inhibitory effect of ethanolamine on CqsR could be a possible source of QS interference but is masked by the presence of the other parallel QS pathways, allowing *V*. *cholerae* to robustly colonize the host.

## Author summary

Many pathogens use quorum sensing (QS) to regulate virulence gene expression for their survival and adaptation inside hosts. QS depends on the production and detection of chemical signals called autoinducers made endogenously by the bacteria. However, chemicals present in the surrounding environment could potentially lead to quorum signal interference, resulting in mis-regulation of virulence factor production and preventing effective host colonization. We show here ethanolamine, a metabolite commonly found inside the mammalian intestine, modulates the activity of one of the QS receptors in *Vibrio cholerae*, the etiological agent of the disease cholera. Despite the abundance of this common metabolite inside the host, by integrating multiple parallel signal inputs into its QS system, *V*. *cholerae* has evolved to maintain QS fidelity and avoids signal interference to allow robust colonization of the host.

## Introduction

**Fig 1:**
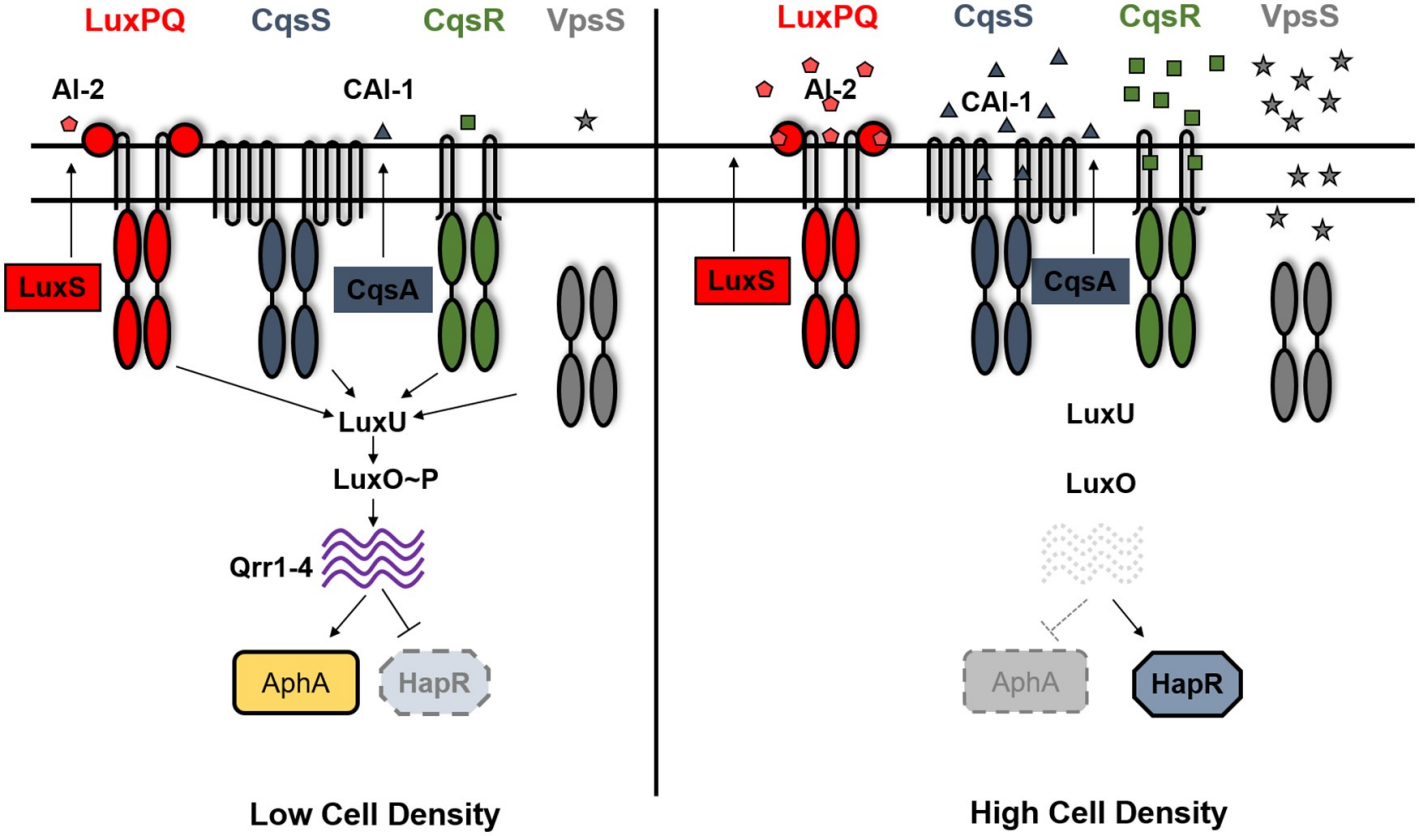
Quorum-sensing circuit in *Vibrio cholerae*. Quorum sensing in *V. cholerae* is controlled by four receptor histidine kinases CqsS, LuxPQ, CqsR and VpsS. At low cell density, these receptors act predominantly as kinases and phosphorylate LuxO through LuxU. Phosphorylated LuxO activates transcription of small RNAs Qrr1-4 which inhibit HapR translation and promote AphA translation, thereby resulting in a low cell density expression profile. At high cell density, when the cognate signals are bound, the kinase activity of these receptors is inhibited. This leads to dephosphorylation of LuxO, preventing Qrr1-4 transcription. Therefore, HapR translation is induced and AphA translation repressed, leading to a high cell density gene expression pattern. Autoinducers CAI-1 (produced by CqsA) and AI-2 (produced by LuxS) have been previously characterized in regulating the kinase activity of CqsS and LuxPQ respectively.

Quorum sensing (QS) is used by a wide variety of bacteria to coordinate population-wide changes in behaviors in response to cell density [1]. The Gram-negative bacterium *Vibrio cholerae*, which causes the diarrheal disease cholera in the human host, uses QS to regulate virulence factor production, biofilm formation, Type VI secretion, metabolic regulation, and natural competence to maintain competitive fitness in various environmental niches [2–11]. Four parallel QS signaling systems have been identified in *V*. *cholerae* that rely on a histidine kinase phosphorelay to regulate downstream gene expression [12] (Fig 1). At low cell-density (LCD), CqsS and LuxPQ function in parallel with two other histidine kinases, called CqsR and VpsS, to phosphorylate LuxO through an intermediate phosphotransfer protein LuxU [10, 12]. Phosphorylated LuxO promotes the transcription of small RNAs Qrr 1–4 which promote the translation of master regulator AphA [13, 14]. Conversely, Qrr 1–4 repress the translation of transcriptional regulator HapR at LCD [15, 16] (Fig 1). At high cell-density (HCD), each receptor kinase detects the presence of unique chemical messengers called autoinducers (AIs) that inhibit the kinase activity of these receptors upon signal binding [1]. Autoinducer synthase CqsA catalyzes the production of CAI-1 (*S*-3-hydroxytridecan-4-one) which is detected by receptor CqsS [17–21] and autoinducer synthase LuxS produces AI-2 (*S*-TMHF-borate) which is detected by LuxP/Q [22–26]. Thus, at HCD, when receptors are bound to their cognate signals, kinase activity is inhibited leading to dephosphorylation of LuxO. This prevents the transcription of Qrr 1–4 thereby inhibiting AphA production and promoting HapR translation.

The kinase activity of both CqsR and VpsS is cell-density dependent but is not controlled by CAI-1 and AI-2, which suggests that *V*. *cholerae* produces unique chemical signals that bind to CqsR and VpsS respectively [12]. Yet, the AI signals that control the activity of CqsR and VpsS have not been identified [12]. VpsS has no predicted transmembrane domains and is therefore thought to be cytoplasmic. Nitric oxide (NO) has been shown to regulate the kinase activity of VpsS *in vitro* through a signaling partner VpsV (VcNosP) [27]. However, *V*. *cholerae* does not make NO and Δ*vpsV* mutants has no detectable change in QS response [10, 12], so the exact role of NO sensing by VpsV in *V*. *cholerae* QS response remains unknown. Another QS circuit was recently identified in *V*. *cholerae*, consisting of a cytoplasmic receptor VqmA and its cognate signal DPO (3,5-dimethylpyrazin-2-ol) but this system does not participate in regulating LuxO [28]. In contrast, CqsR is predicted to have a periplasmic ligand binding domain that presumably is involved in signal detection and influences the activity of the cytoplasmic histidine kinase domain.

Since any one of the QS receptors is sufficient to activate LuxO to support *V*. *cholerae* colonization in the small intestine of the host, it is puzzling that four parallel circuits are converged to control a single regulator to regulate its QS response [12]. It has been proposed that such network architecture could avoid signal interference on any one of the QS receptors, thus, a robust QS response can be maintained even in the presence of other signal noise that targets one of the four inputs [12]. However, it is unclear what natural chemicals, other than the natively produced AIs, can change the activity of these QS receptors and interfere with the *V*. *cholerae* QS response. Moreover, environments in which QS interference would be detrimental to the fitness of this pathogen have not been well studied. In this study, we probe these two questions further and show that mutants expressing only CqsR without the other three QS receptors are defective in colonizing the host large intestine, a niche where wild-type *V*. *cholerae* colonizes effectively and is relevant to asymptomatic carriage of the pathogen [29, 30]. We developed a chemical binding assay and identified ethanolamine, a common intestinal metabolite, as a ligand for CqsR. Using whole cell QS reporter assays and the infant mouse model, we show that ethanolamine can function as a chemical source of QS interference on CqsR. However, *V*. *cholerae* has evolved to avoid signal interference by ethanolamine by incorporating four parallel pathways into its QS system, and this specific circuit architecture enables robust colonization of the host.

## Results

### Aberrant QS signaling leads to decreased *V*. *cholerae* colonization in the large intestine

**Fig 2:**
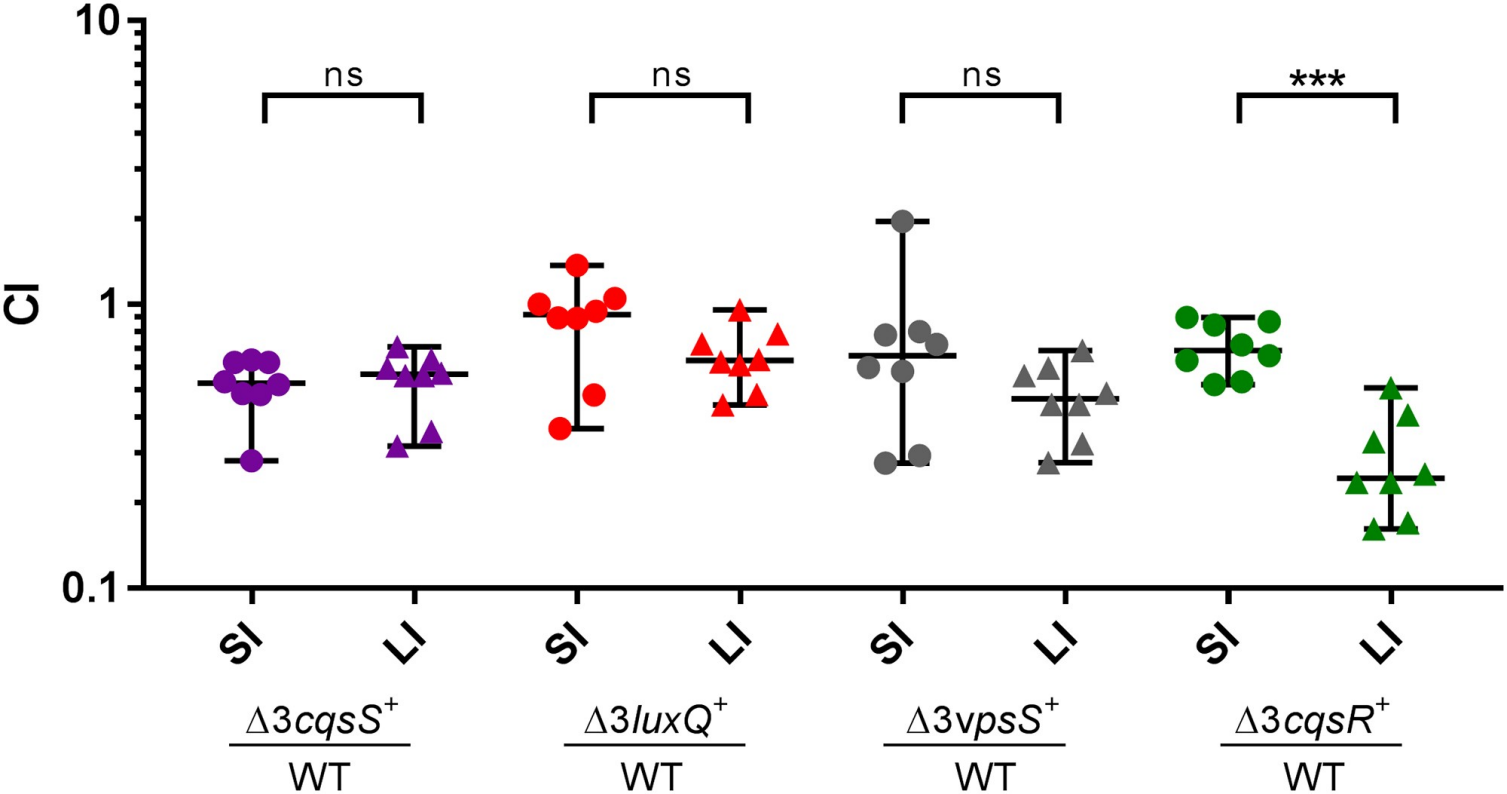
Effect of QS receptor mutations in *V*. *cholerae* infection of the small and large intestine. Competitive indices (CI) were determined between wild-type Δ*lacZ* and the indicated *V*. *cholerae* mutants in the small intestine (SI) and large intestine (LI) of infant mice 24 hr post-infection (n = 8). Δ3 represents triple receptor mutants with the remaining receptor shown in italics. Each symbol represents the CI in an individual mouse and data is represented with horizontal lines indicating the median with a 95% confidence interval for each competition. ***P < 0.001; ns = no significance (unpaired t test).

We previously showed that any one of the QS receptors was sufficient to promote response regulator LuxO activation and support *V*. *cholerae* colonization of the mouse small intestine [12]. In contrast to the small intestine, the large intestine has higher microbial loads and possibly increased potential for QS signal interference [31, 32]. Previous studies have shown that both El Tor and classical biotypes colonize the large intestine in mouse models, suggesting that this niche could be relevant for human infections, especially in asymptomatic carriers and some convalescing patients [29, 30]. Therefore, we compared the four triple (Δ3, missing any three of the four QS receptors) and the quadruple (Δ4, missing all four QS receptors) receptor mutants with the wild type for their ability to colonize both the small and large intestines using the infant mouse colonization model. As reported previously, all four Δ3 mutants colonize the small intestines comparable to the wild type (Fig 2) while the Δ4 mutants are highly defective for colonization [12]. However, the colonization pattern in the large intestine of these Δ3 receptor mutants is different. Specifically, the triple receptor mutants expressing only CqsR (Δ3 *cqsR*^*+*^) are significantly less efficient in colonizing the large intestine than in the small intestine, while the other three Δ3 mutants are not significantly different in colonizing these two intestinal sites (Fig 2). Colonization of the large intestine is dependent on LuxO activation and downstream virulence factor production since the Δ4 mutants and the mutants missing the major toxin co-regulated pilus (Δ*tcpA*) are unable to colonize this site (S1 Fig). Our findings suggest that a previously uncharacterized factor is present in the large intestine but not in the small intestine, and this factor acts specifically on CqsR and lowers the kinase activity of the receptor. Without the other three QS receptors to compensate for LuxO activation, this interfering factor could lead to repression of virulence gene expression, resulting in a defect of the Δ3 *cqsR*^*+*^ mutants in colonization this specific region of the gastrointestinal (GI) tract.

### Identification of residues in the CqsR periplasmic region that are important for signal sensing or signal transduction

**Fig 3:**
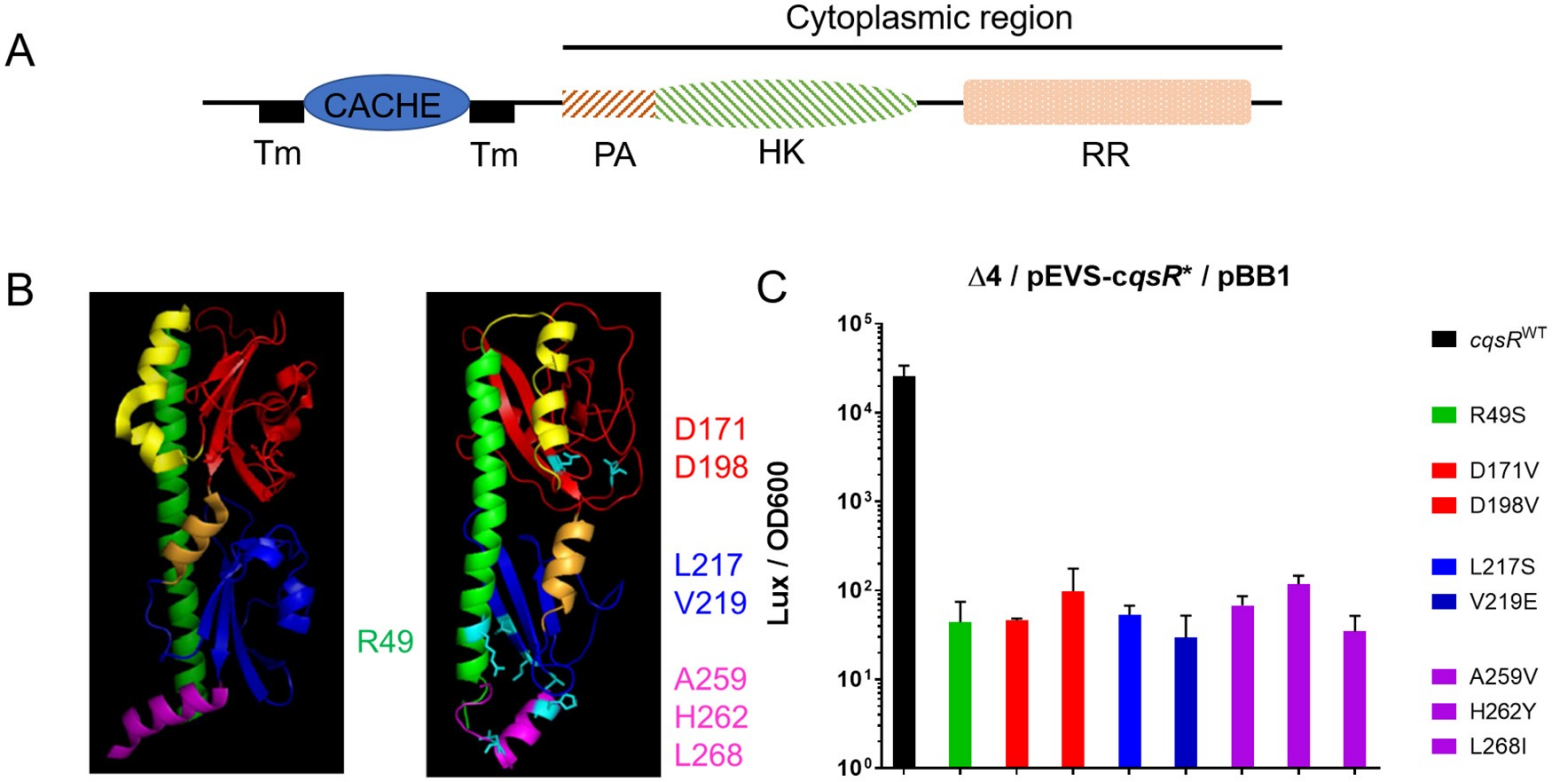
The CqsR CACHE domain is important for signal sensing and signal transduction. (A) CqsR is predicted to contain all the conserved cytoplasmic domains (phosphoacceptor (PA), histidine kinase (HK), and response regulator (RR) domains) of a hybrid histidine kinase with an N-terminal periplasmic CACHE domain flanked by two transmembrane helices (TM). (B) The predicted structure of the periplasmic CACHE domain of CqsR (right) is similar to that of Mlp37 (left). The residues identified as important for signal sensing and signal transduction are highlighted in cyan in the predicted structure. See main text for further details. (C) Eight periplasmic CqsR residues are involved in signal sensing and signal transduction. Relative light production (lux/OD_600_) was measured in quadruple QS receptor (Δ4) strains carrying a plasmid producing CqsR with single amino changes as well as a HapR-dependent bioluminescence reporter. Alteration in the CqsR helix domain (R49S), CACHE pocket 1 region (D171V, D198V), CACHE pocket 2 region (L217S, V219E), or the transmembrane proximal regions (A259V, H262Y, L268I) impaired bioluminescence production at high cell-density (OD_600_ > 1.5). Average values and standard errors from at least three independent replicates are shown.

Using various bioinformatics tools, the periplasmic region of CqsR was predicted to carry a periplasmic dCACHE_1 domain ([33, 34], residues 44–239, simplified as CACHE domain hereafter) (Fig 3A) that displays structural features similar to other CACHE domains, such as the signal sensing domain of *V*. *cholerae* chemoreceptor Mlp37 [35] (Fig 3B). The predicted CqsR periplasmic region contains the characteristic long N-terminal α-helix (green), a linker (yellow) that connects this helix to two globular domains (pocket I, red and pocket II, blue) with a small α-helical linker between the two pockets (orange), and a C-terminal α-helix (purple) that connects the domain to the cytoplasm (Fig 3B). These similarities strongly suggest that the periplasmic region of CqsR functions as a ligand binding domain to regulate its cytoplasmic kinase activity. We constructed and screened a *cqsR* plasmid library in which the whole periplasmic region was randomly mutagenized to identify mutations in this domain that impair signal sensing and turn CqsR into a constitutively active kinase even in the presence of the cognate signal above the threshold detection level. The randomly mutagenized *cqsR* library was introduced into a quadruple receptor mutant (Δ4) *V*. *cholerae* strain that also harbors a HapR-dependent bioluminescent reporter cosmid pBB1 [12]. While the quadruple receptor mutant produced light constitutively due to the constant production of HapR, when a wild-type (WT) copy of *cqsR* was introduced, this strain produced low bioluminescence at low cell-density (LCD) and subsequently turned on the production of bioluminescence at high cell-density (HCD) due to signal detection and kinase inhibition of CqsR [12]. Thus, while clones carrying plasmids expressing WT *cqsR* or a null *cqsR* allele would appear bright at HCD, clones producing CqsR variants insensitive to the presence of cognate signal would produce lower bioluminescence at HCD. We measured the bioluminescence of ~26,000 clones from this library and identified 79 candidates impaired for light production at HCD. Most candidates harbored multiple mutations in the plasmid-borne *cqsR*. There were 23 unique mutations in these candidates. When each unique mutation was introduced back to *cqsR* individually, only 8 unique mutations in *cqsR* resulted in lower bioluminescence production at HCD (Fig 3C). These *cqsR* mutations caused changes in the two aspartate residues (D171V and D198V) mapped to pocket I of the predicted ligand binding domain, two changes in pocket II (L217S and V219E), four changes (R49S, A259V, H262Y, and L268I) in the two helical regions that either exit or enter the cellular membrane and are likely not involved in direct ligand interactions. Notably, both Asp171 and Asp198 residues in the pocket I of CqsR are predicted to be in the same positions as the Asp172 and Asp201 residues respectively, in Mlp37, which have been shown to be critical for binding to amino acid signals [35]. Therefore, our results suggest that, similar to Mlp37, CqsR detects its cognate signals through the periplasmic CACHE domain.

### Chemical screen identified ethanolamine as a potential CqsR ligand

**Fig 4:**
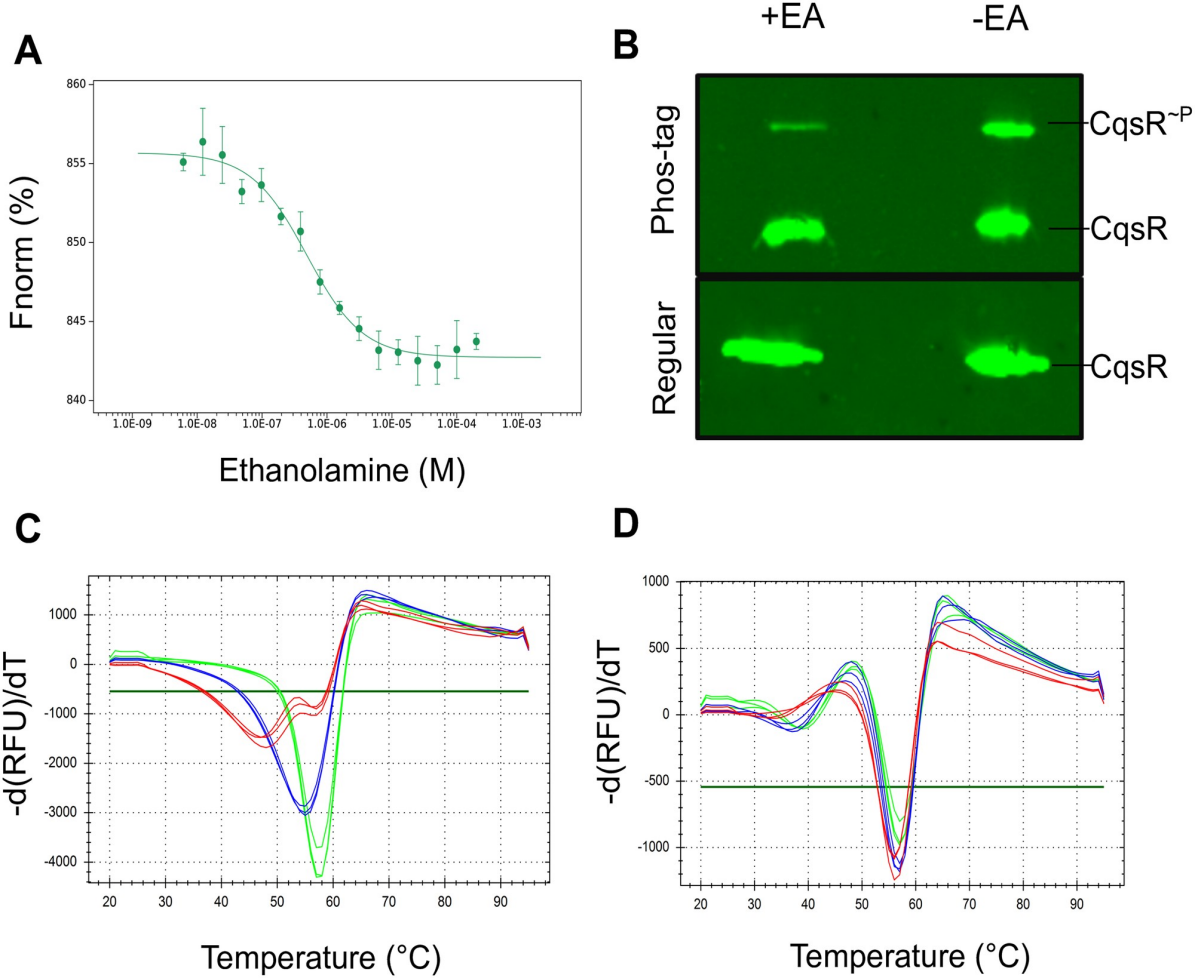
Characterization of ethanolamine binding to CqsR. (A) MST quantification (FNorm; normalized fluorescence) for ethanolamine binding to CqsR was performed by titrating between 200 μM and 0.0061 μM with 20 nM His6-tagged CqsR. Ethanolamine binds to CqsR with a Kd of 0.478 ± 0.076 μM. Binding affinity was calculated from three independent experiments. B) Immunoblots of CqsR using Phos-tag (top) and regular gels (bottom). The singly phosphorylated CqsR runs as a distinct band at a higher molecular weight compared to the unphosphorylated CqsR with a Phos-tag gel. The ratio of phosphorylated to unphosphorylated forms of CqsR is substantially lower in the presence of 10 mM ethanolamine. Differential scanning fluorimetry melt curves of purified C) MBP-CqsR-LBD and D) MBP-CqsR^D171V^-LBD in the presence of 0 μM (red), 20 μM (blue), 200 μM (green) ethanolamine. A positive shift in Tm of MBP-CqsR-LBD indicates ligand binding, while no shift is observed for MBP-CqsR^D171V^-LBD in the presence of ethanolamine.

Having confirmed that the periplasmic CACHE domain is critical for signal sensing, we then attempted to identify potential ligands capable of binding to this putative CqsR ligand binding domain (CqsR-LBD), by performing a differential scanning fluorimetry chemical screen using the common metabolites contained within the Biolog^™^ PM1-4 plates. This assay assumed that ligand binding leads to stabilization of the target protein, resulting in an increase of melting temperature (Tm) of the protein, which can be determined by measuring the dynamics of Sypro Orange fluorescence emission at different temperatures. In the absence of exogenous metabolites, the average Tm of the purified CqsR-LBD was observed to be 46 °C under the test conditions. Out of the ~400 metabolites tested, including some of the known compounds that bind CACHE domain such as amino acids, carboxylates, and polyamines, we only observed a shift in the Tm of >5 °C in the presence of one tested compound, ethanolamine (or 2-aminoethanol). The shift in Tm of CqsR in the presence of ethanolamine was dose-dependent. The largest shift in Tm of +15 °C was observed for 10 mM ethanolamine (Table 1) with smaller shifts of +9.33 °C, +6°C and +2°C for 1 mM, 100 μM and 10 μM ethanolamine respectively. Using MicroScale Thermophoresis, we determined the binding affinity of ethanolamine to CqsR-LBD to be around 0.5 μM (Fig 4A).

**Table 1:** Effects of ethanolamine and its analogs on the melting temp of CqsR-LBD.

Compound^a^	Tm (°C)^b^
None	46
Ethanolamine [2-aminoethanol]	61
Cysteamine [2-aminoethanethiol]	46
1-propanamine	46.33 [1.15]
β-alanine [2-Carboxyethylamine]	46
Mercaptoethanol [2-Hydroxyethanethiol]	46
1-Propanol	43.67 [0.58]
2-Methylaminoethanol	49.67 [0.58]
2-Dimethylaminoethanol	48
Choline [(2-Hydroxyethyl)trimethyl ammonium]	46
(S)-(+)-1-Amino-2-propanol	48.67 [0.58]
(R)-(−)-1-Amino-2-propanol	48
L-alaninol [(S)-(+)-2-Amino-1-propanol]	60
D-alaninol [(R)-(−)-2-Amino-1-propanol]	45
Serinol [2-Amino-1,3-propanediol]	53.67 [0.58]

^a^ Structure of each compound is shown in S3 Table

^b^ Data shown as average of 3 replicates in the presence of 10 mM tested compounds. Square brackets indicate standard deviation where applicable.

We then determined how chemical modifications to the ligand affect CqsR-LBD binding (Table 1). Replacing the hydroxyl group of ethanolamine with a thiol, methyl, or carboxyl group abolished *in vitro* binding to CqsR-LBD, as indicated by the lack of change in the observed Tm in the presence of these tested compounds. Similarly, replacing the amine group with a thiol or methyl group also abolished binding (Table 1). In addition, single or multiple methylation of the amine group of ethanolamine also abolished binding (Table 1). We therefore concluded that the presence of both the amine and the hydroxyl groups in ethanolamine are essential for efficient CqsR-LBD binding. Side-chain substitutions at the carbon atom next to the hydroxyl group also abolished CqsR-LBD binding regardless of the stereochemistry of the hydroxyl group. However, a limited number of substitutions on the carbon atom next to the amine group do not affect CqsR binding, as indicated by the increase in Tm observed for L-alaninol and serinol (Table 1). Interestingly, stereospecificity is critical for binding activity as indicated by lower shift in Tm for D-alaninol, a stereo-isomer of L-alaninol, at the same concentration (Table 1).

A recent study showed that a specific ethanolamine derivative, N-(2-hydroxyethyl)-2-(2-hydroxyethylamino) acetamide [HEHEAA], present in commercial preparations of ethanolamine, is responsible for promotion of *pipA* transcription in *Pseudomonas sp*. *GM79* in response to plant exudates [36]. We tested eight common compounds that accumulate during chemical synthesis of ethanolamine including HEHEAA [36, 37] for their ability to bind to CqsR and, in our hands, none of these compounds binds to CqsR-LBD (S2 Table). Collectively, our data indicate that only ethanolamine and a limited number of its derivatives bind to CqsR-LBD with a high degree of specificity.

To test whether the presence of ethanolamine affects autophosphorylation of CqsR, we over-expressed full-length His_6_-CqsR in *E*. *coli* cells grown in the absence and presence of 10 mM ethanolamine and measured the extent of CqsR autophosphorylation using Phos-Tag gel electrophoresis [38] (Fig 4B). As expected, CqsR ran as a single band in the absence of Phos-Tag, in contrast, phosphorylated CqsR was separated from the unphosphorylated CqsR in the gel containing Phos-Tag. Although CqsR is a hybrid histidine kinase where two phosphorylation sites are present, we only detected singly phosphorylated CqsR under this condition (Fig 4B). In the absence of exogenous ethanolamine, the ratio of phosphorylated to unphosphorylated species of CqsR was 0.53:1 which decreased to 0.07:1 when 10 mM ethanolamine was added to the medium, (Fig 4B). Our results suggest that ethanolamine binding to CqsR reduces its auto-phosphorylation activity.

We also tested whether CACHE domain mutations in CqsR affects ethanolamine binding. As His_6_-tagged CqsR^D171V^-LBD is insoluble and expressed poorly in *E*. *coli*, we used maltose binding protein (MBP) tagged CqsR^WT^-LBD and CqsR^D171V^-LBD in the thermal shift assay. Ethanolamine bound to the purified MBP-CqsR^WT^-LBD as evidenced by positive shifts in Tm of +8 °C and +12°C in the presence of 20 uM and 200 uM ethanolamine respectively (Fig 4C), however no Tm shift was observed for purified MBP-CqsR^D171V^-LBD under the same conditions (Fig 4D). These data suggest that mutations in the CACHE domain prevents ethanolamine sensing by CqsR.

### Effect of ethanolamine and its analogs in regulating *V*. *cholerae* quorum-sensing activity

**Fig 5:**
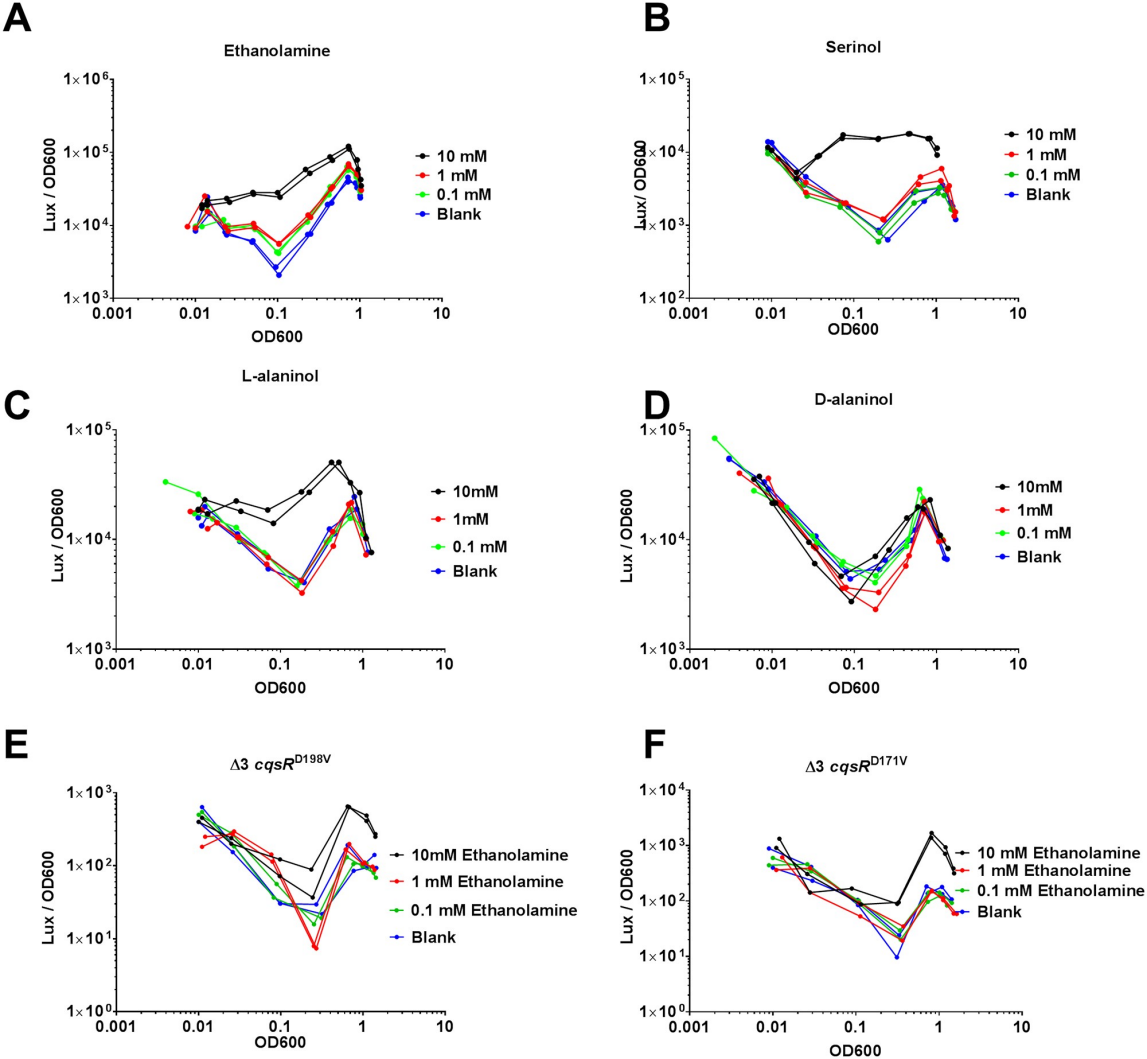
Effects of ethanolamine and its analogs on CqsR quorum-sensing response. HapR-dependent bioluminescence profiles (lux/OD_600_) were measured in a Δ3 *cqsR*^+^ strain in the presence of 10 mM, 1mM, 0.1 mM A) ethanolamine, B) serinol, C) L-alaninol, D) D-alaninol. Blank indicates LB medium without ethanolamine added. HapR-dependent bioluminescence profiles (lux/OD_600_) were measured in E) Δ3 *cqsR*^D198V^ strain and F) Δ3 *cqsR*^D171V^ strain in the presence of 10 mM, 1mM, 0.1 mM concentrations ethanolamine respectively. Each figure shows a representative profile of each condition with two biological replicates. Each experiment was performed independently at least two times.

Since ethanolamine binds to the CqsR-LBD *in vitro* and decreases autokinase activity of CqsR, we then determined the effect of exogenous addition of ethanolamine on the induction of premature HCD QS response in *V*. *cholerae*. We measured the density-dependent bioluminescence profile of a Δ3 *cqsR*^+^ strain that harbors the HapR-dependent bioluminescent reporter pBB1 in the presence of varying concentrations of exogenously added ethanolamine. In the absence of ethanolamine, similar to previously reported [12], the Δ3 *cqsR*^+^ strain displayed the characteristic U-shaped HapR-dependent bioluminescence profile due to changes in CqsR kinase activity in different cell densities (Fig 5A). However, we saw increased HapR-dependent light production in the presence of ethanolamine, indicating cells prematurely induced a HCD-like QS state. This response was dose-dependent. (Fig 5A). In the presence of 10 mM exogenously added ethanolamine, the strain was constitutively bright at all cell densities and produced ~10 fold more bioluminescence at LCD when O.D. ~0.1 when compared to that from the culture in LB medium without ethanolamine added (Fig 5A). Moreover, lower concentrations (1mM or 0.1mM) of exogenous ethanolamine also induced a higher level of light production in this reporter strain. Similar results were obtained for constitutive bioluminescence induction in the presence of L-alaninol and serinol at 10 mM (Fig 5B and 5C). However, 10 mM D-alaninol was inactive, consistent with the observation that D-alaninol did not shown any detectable binding activity to CqsR (Table 1, Fig 5D). A reciprocal pattern was observed for P*_qrr_*_4_-*lux* expression, where 10mM ethanolamine, serinol and L-alaninol, but not D-alaninol, were able to repress Qrr sRNA transcription in the Δ3 *cqsR*^+^ strain (S2 Fig). In addition, ethanolamine did not induce HapR-dependent bioluminescence in other triple QS receptor mutants where only CqsS, LuxPQ, or VpsS is present as the sole QS receptor (S3 Fig), indicating that ethanolamine induces QS in a CqsR specific manner. Finally, unlike the Δ3 strain producing WT CqsR, 10 mM of ethanolamine did not induce constitutive light production in the Δ3 CqsR^D171V^ and Δ3 CqsR^D198V^ CACHE domain mutants (Fig 5E and 5F). Together, our results strongly suggest that ethanolamine specifically interacts with the CACHE domain of CqsR to inhibit its kinase activity and modulate QS gene expression in *V*. *cholerae*. Interestingly, ethanolamine also caused a small but reproducible decrease of light production in Δ3 *vpsS*^+^ but the mechanism is currently unknown.

### Ethanolamine can modulate CqsR QS signaling inside animal hosts

**Fig 6:**
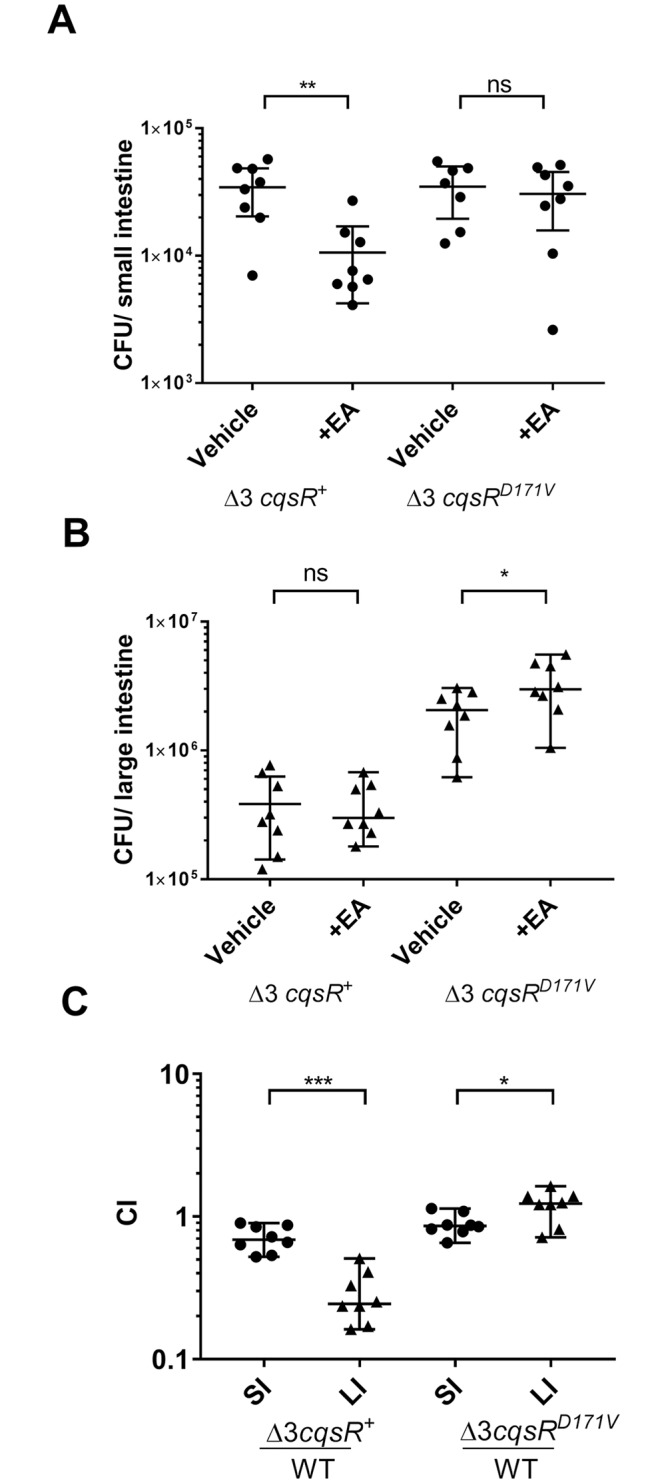
Effect of ethanolamine on CqsR quorum-sensing response inside animal hosts. CFU counts per A) small intestine homogenate or B) large intestine homogenate collected 8 hr post infection from mice (n = 8) singly infected with a Δ3 *cqsR*^+^ or Δ3 *cqsR*^D171V^ mutant strain. To assess the effect of ethanolamine on strain colonization, mice were gavaged with vehicle (LB) or 10mM ethanolamine (EA) at 2 and 4 hours post infection. C) Competitive indexes (CI) were determined between wild-type Δ*lacZ* and the indicated *V*. *cholerae* mutants in both the small intestine (SI) and large intestine (LI) of infant mice 24 hr post infection (n = 8). The Δ3 *cqsR*^+^ data presented in Fig 2 has been included for reference and comparison to Δ3 *cqsR*^D171V^. Each symbol represents the CI in an individual mouse and data is represented with horizontal lines indicating the median with a 95% confidence interval for each group. *P < 0.05; **P < 0.01; ***P < 0.001 (unpaired t test). n.s. = not significant.

Since the production of TcpA, the major colonization factor, is repressed by HCD [5, 6], we assayed whether ethanolamine would prematurely induce a HCD-like state *in vivo* and affect the ability of Δ3 *cqsR*^+^ and Δ3 *cqsR*^D171V^ strains to colonize infant mice. We predicted that Δ3 *cqsR*^+^ would have a lower efficiency in colonizing the GI tract, while the Δ3 *cqsR*^D171V^ mutant would be unaffected due to its insensitivity to exogenous ethanolamine. Indeed, in the absence of exogenous ethanolamine, the Δ3 *cqsR*^+^ strain colonized the infant mouse small intestine with the same efficiency as the Δ3 *cqsR*^D171V^ strain, which does not detect ethanolamine (Fig 6A). However, in the presence of exogenous ethanolamine, the Δ3 *cqsR*^+^ strain showed a ~5-fold decrease in the colonization of the small intestine compared to the Δ3 *cqsR*^D171V^ strain (Fig 6A). Addition of exogenous ethanolamine however, did not negatively impact colonization of either the Δ3 *cqsR*^+^ or the Δ3 *cqsR*^D171V^ strain within the large intestine. This is likely due to the orally gavaged ethanolamine could not reach the large intestine, or due to saturation of the signal already present in that site (Fig 6B). We also compared colonization of the Δ3 *cqsR*^+^ and the Δ3 *cqsR*^D171V^ strains in competition with WT *V*. *cholerae*. While the Δ3 *cqsR*^+^ showed a ~5-fold defect in colonizing the large intestine, the Δ3 *cqsR*^D171V^ could colonize both the small and the large intestines equally well (Fig 6C, S4A and S4B Fig). Despite these differences in colonization, we did not observe significant differences in *tcpA* expression in different strains colonized in the large intestine (S4C Fig). It is likely that bacteria with decreased *tcpA* expression level due to ethanolamine interference on CqsR were incapable of colonizing this niche and not sampled; and the colonized bacteria that were sampled were the ones that did not experience ethanolamine interference. Finally, we measured the total amount of ethanolamine present in both the small and large intestinal environments using LC/MS-MS. The total ethanolamine content was determined to be an average of 0.8±0.3 ng and 0.2±0.1 ng of ethanolamine, respectively, in the small and large intestine of the suckling mouse. However, since the luminal surface of the small intestine ranges between 9–20 times larger than that of the large intestine [39, 40]. After correcting for the difference in surface area, ethanolamine surface density was estimated to be 2 to 4 fold higher in the large intestine than the small intestine (S4D Fig). Taken together, our results suggest that ethanolamine, among other metabolites present in the large intestine, inhibits the kinase activity of CqsR and lowers the colonization efficiency of the Δ3 *cqsR* mutant, but this interference is masked by the action of other QS receptors in the wild type. Consistent with this finding, we also observed that while exogenous ethanolamine induced a premature HCD QS response in Δ3 *cqsR*^+^ strain (i.e. increased bioluminescence), no premature induction was observed *in vitro* in WT *V*. *cholerae* due to the masking by other receptors (S5 Fig).

### The role of intrinsically produced ethanolamine in QS

We then tested if endogenously made ethanolamine also plays a role in controlling CqsR activity. In *E*. *coli*, ethanolamine is generated as a byproduct of glycerophospholipid metabolism, derived from the degradation of glycerol-3-phosphoethanolamine to glycerol-3-phosphate and ethanolamine [41, 42]. This reaction is predicted to be catalyzed by two *V*. *cholerae* glycerophosphodiester phosphodiesterases VCA0136 (GlpQ homolog) and VC1554 (UgpQ homolog). We therefore deleted the genes encoding both phosophodiesterases in the Δ3 *cqsR*^+^ strain. ^1^H NMR was used to measure ethanolamine level in the cell-free supernatants derived from both the Δ3 *cqsR*^+^ as well as the Δ3 *cqsR*^+^ Δ*vca0136* Δ*vc1554* strain grown in LB medium. We detected ~100 μM ethanolamine in LB medium alone, which was used as a baseline to determine ethanolamine produced by the two strains. Supernatants from Δ3 *cqsR*^+^ contained a net of ~25 μM ethanolamine while no additional ethanolamine was detected in the supernatants from the Δ3 *cqsR*^+^ Δ*vca0136* Δ*vc1554*. Similarly, supernatants from Δ3 *cqsR*^+^ grown in M9 + 1% casamino acids contained 40 μM ethanolamine while the Δ3 *cqsR*^+^ Δ*vca0136* Δ*vc1554* mutants contained no detectable ethanolamine.

**Fig 7:**
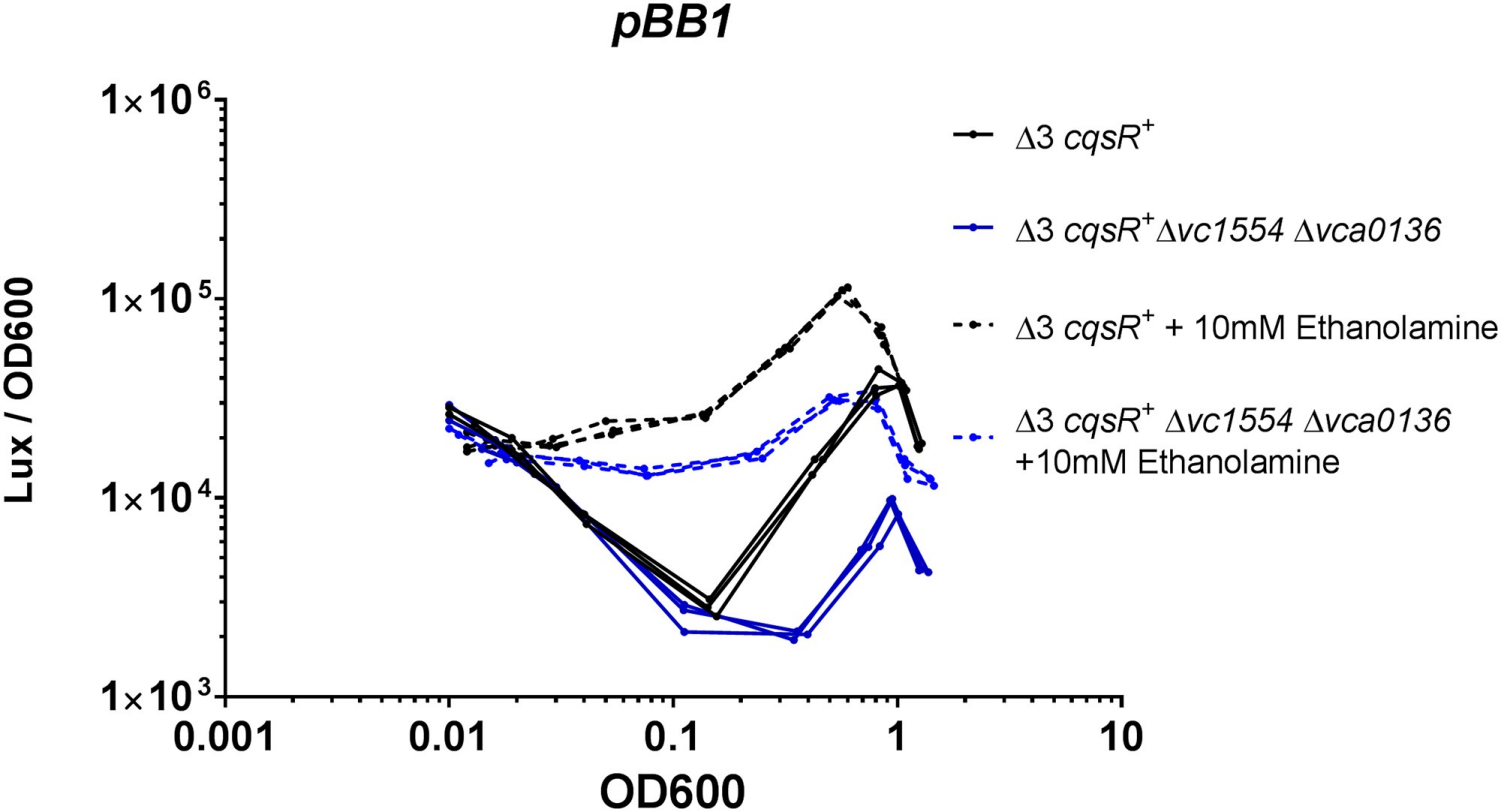
Effect of glycerophosphodiesterase deletions on CqsR quorum-sensing response. HapR-dependent bioluminescence profiles (lux/OD_600_) were measured in a Δ3 *cqsR*^+^ strain and an isogenic strain with deletions in loci *vc1554* and *vca0136*, encoding the two putative glycerophosphodiester phosphodiesterases in LB medium and in the presence of 10 mM ethanolamine. The figure shows a representative profile of each condition with two biological replicates. Each experiment was performed independently at least two times.

After confirming the mutants did not make any ethanolamine, we used the HapR-dependent bioluminescence pBB1 reporter to measure the QS response of these strains. Similar to the Δ3 *cqsR*^+^ strain, the isogenic double phosphodiesterase deletion mutants still exhibited density-dependent U-shaped bioluminescence profile. Nevertheless, the onset of the transition to HCD bioluminescence production was slightly delayed in the double phosphodiesterase mutants (Fig 7). Moreover, addition of 10 mM ethanolamine increased light production in Δ3 *cqsR*^+^ as well as in the Δ3 *cqsR*^+^ Δ*vca0136* Δ*vc1554* carrying the pBB1 HapR-dependent reporters (Fig 7), indicating CqsR sensing is functional in responding to exogenously supplied ethanolamine in these strains. Consistent with the above results, the reciprocal expression pattern was observed using a P*_qrr_*_4_-*lux* reporter (S6A Fig). Our results indicate that endogenously made ethanolamine only partially participates in QS gene regulation. It should be noted that, even in the absence of endogenous ethanolamine production, light production increased at higher cell density in the Δ3 *cqsR*^+^ Δ*vca0136* Δ*vc1554* strains (Fig 7), indicating *V*. *cholerae* makes other signals that inhibit CqsR kinase activity at HCD. Consistent with this idea, supernatants from overnight cultures of the either the Δ3 *cqsR*^+^ or Δ3 *cqsR*^+^ Δ*vca0136* Δ*vc1554* equally inhibited *qrr*4-*lux* expression (S6B and S6C Fig).

Ethanolamine is particularly abundant in the GI tract of large mammals, where it can be found at concentrations over 2 mM per some reports [43]. Some, but not all, enteric pathogens that sense ethanolamine as a signal are also capable of metabolizing ethanolamine as a nutrient source [43–46]. *V*. *cholerae* lacks the canonical *eut* operon for ethanolamine metabolism [47] and we confirmed that the bacteria cannot metabolize ethanolamine either as carbon or nitrogen source (S7 Fig). Therefore, CqsR response to ethanolamine is likely not for nutrient sensing.

## Discussion

The presence of four parallel QS signaling pathways in *V*. *cholerae* is perplexing since detection of multiple signals to control a single regulator would appear to be functionally redundant. We previously suggested that the major advantage of this “many-to-one” circuit arrangement is to prevent signal perturbation and to increase the robustness of the system so that the HCD QS response is only expressed when all four receptors are bound with the cognate signals [12]. Our findings in this study further support this idea. Importantly, we have identified the host large intestine as a specific niche where this unique QS network architecture is essential for the fitness of the pathogen. It is reasonable that mutants with an aberrant QS circuit are defective only in colonizing the large intestine, a niche that is relevant to the asymptomatic carriage of the *V*. *cholerae* [29, 30] and has a significantly higher bacterial load and contains a more complex bacterial community [31, 32]. Indeed, intestinal pathogens have adopted various strategies to overcome the challenges caused by chemical interferences encountered in this complex environment [48, 49]. We show that although the common intestinal metabolite ethanolamine and likely other chemicals present in the large intestine can interfere with CqsR signaling, *V*. *cholerae* has evolved to withstand the interference by having multiple QS inputs for effective host colonization. This kind of network architecture was previously proposed to be a coincidence detector [50, 51] where the QS system is finely-tuned to enable transition to HCD only in the most appropriate environment. Indeed a recent report suggested that QS-dependent biofilm disassembly in *V*. *cholerae* operates as also a coincidence detector, where Vibrio specific (CAI-1) as well as the universal bacterial signal AI-2 need to be detected concurrently before cells can transition from low cell density to high cell density to disrupt biofilm formation [52]. The interaction of *V*. *cholerae* QS response with other chemicals present inside the GI environment is not limited to just ethanolamine, for instance, certain microbiota species can affect *V*. *cholerae* virulence in an AI-2 dependent but LuxP independent manner [53]; and the presence of certain microbiota species appears to correlate with the susceptibility of *V*. *cholerae* infection [54].

It should be noted that signals that regulate the activity of most histidine kinases are often unknown, here we demonstrate that ethanolamine acts as a direct ligand that inhibits the CqsR kinase activity in *V*. *cholerae*. Although we cannot rule out molecules present in the GI tract other than ethanolamine can also act on CqsR, other enteric pathogens including *E*. *coli*, *Salmonella*, *Enterococcus*, and *C*. *difficile* have adopted different mechanisms to sense this common metabolite to regulate their gene expression to improve their fitness inside the host [44, 46, 55–58]. Some bacteria also sense ethanolamine outside of the GI tract to regulate gene expression [44, 59–62]. In addition, previous studies have demonstrated that ethanolamine utilization can both positively and negatively affect host colonization and virulence during infection [44, 46, 55, 59–63]. We and others [47], showed that certain *Vibrios* are unable to metabolize ethanolamine as either carbon or nitrogen source, nevertheless, the influence of ethanolamine on virulence gene expression can be independent of ethanolamine metabolism [44, 57].

We postulate that ethanolamine acts specifically on CqsR for multiple reasons. First, CqsR could serve as a dual-function receptor that senses an uncharacterized self-made autoinducer to monitor cell density and an exogenous metabolite such as ethanolamine as an environmental cue. This kind of dual detection of cognate AIs and other chemical signals by a single QS receptor is quite unusual. QseC is one of the known QS receptors that detects both an AI signal (AI-3) and other chemical cues (epinephrine and norepinephrine) to control virulence gene expression in *E*. *coli* and *Salmonella* [64]. It is proposed that such unique QS receptors can be used for communication between the pathogens and the hosts [64]. Ethanolamine detection through CqsR, together with inhibition of the other QS receptors, would lead to repression of virulence gene expression by promoting HCD QS response in *V*. *cholerae* [12]. This is especially relevant to the life cycle of *V*. *cholerae* since the major colonization site of this pathogen is the small intestine while ethanolamine may be more abundant in the lower GI tract [65], thus, ethanolamine sensing could be used as a proxy for locating different regions in the GI tract. We also cannot rule out that ethanolamine regulates other genes outside of the QS regulon.

In addition, since the mutants defective in ethanolamine synthesis are still able to express a delayed HCD QS response, additional signals must be made by *V*. *cholerae* and detected by CqsR, and these molecules could compete with ethanolamine for binding to CqsR. Therefore, the other possibility is that ethanolamine structurally resembles the cognate signal(s) that CqsR has evolved to detect, so the interactions between CqsR and ethanolamine are just coincidental. The periplasmic CACHE domain is common in many chemotaxis receptors and other membrane bound regulators [34]. CACHE domain proteins have been shown to interact with a variety of chemical compounds and sometimes the same protein can detect multiple structurally similar ligands [33–35, 66–68]. While CACHE domains have been shown to interact with quaternary ammonium compounds such as choline (an ethanolamine analog that CqsR does not interact with) to control chemotaxis [69, 70], our study provided an example where a CACHE domain interacts with an amino alcohol (i.e., ethanolamine) to regulate QS gene expression. Of note, the signal sensing domain of CqsR shares no primary sequence homology to that of another ethanolamine sensing histidine kinase HK17 (EutW) in *Enterococcus faecalis* or the cytoplasmic transcriptional regulator EutR that detects ethanolamine in *Salmonella* [57, 71]. We noticed that although ethanolamine binds to CqsR-LBD with sub-micromolar affinity (k_d_ ~0.5 μM), we needed to supply a higher level of ethanolamine exogenously to induce the HCD QS response in *V*. *cholerae*. This is in stark contrast to the typical effective concentration for canonical AIs, such as CAI-1 and AI-2, which are active at micromolar levels [17–20, 25, 26]. While the exact reason for this difference is not understood, we reasoned that *V*. *cholerae* could have evolved to distinguish the natively made autoinducer signal and exogenous ethanolamine by having different CqsR responses to these two molecules. The chemical interplay between ethanolamine and the cognate signal(s) of CqsR and their roles in *V*. *cholerae* pathogenesis remain to be investigated.

## Materials and methods

### Bioinformatics

Conserved domain structures for the CqsR protein (NCBI Reference Sequence: NP_231465.1) were analyzed using a suite of online tools including CDD (NCBI) [72], CDART(NCBI) [73]and InterPro (EMBL-EBI) [74] while transmembrane regions were predicted using the TMHMM server v2.0 [75]. A map of predicted domains was manually constructed using the outputs generated by these tools as a guide. 3D structural modelling of the CqsR ligand binding domain was performed using HHPred [76] and the Phyre2 algorithm [77] and compared to the known structure of the periplasmic region of Mlp37 (PDB ID: 3C8C) [35].

### Strains, media and culture conditions

All *V*. *cholerae* strains used in this study were derived from C6706*str*2, a streptomycin-resistant isolate of C6706 (O1 El Tor) [78]. Some of the *V*. *cholerae* reporter strains carrying the HapR-dependent bioluminescent reporter pBB1 have been previously described [12]. *V*. *cholerae* and *E*. *coli* cultures were grown with aeration in Luria-Bertani (LB) broth at 30°C and 37°C, respectively. Unless specified, media was supplemented with streptomycin (Sm, 100 μg/ml), tetracycline (Tet, 5 μg/ml), ampicillin (Amp, 100 μg/ml), kanamycin (Kan, 100 μg/ml), chloramphenicol (Cm, 5 μg/ml) and polymyxin B (Pb, 50 U/ml) when appropriate. A complete list of strains used in this study is provided in S1 Table.

### DNA manipulations and strain construction

All DNA manipulations were performed using standard procedures. High-fidelity PCR was performed using Phusion DNA polymerase. Taq DNA polymerase was used for routine screenings. Deletions and point mutations were introduced into the *V*. *cholerae* genome by allelic exchange using the suicide vector pKAS32 (Amp resistant) or a modified version, pJT961 that contains Kanamycin resistance as the counterselection marker [79, 80]. Mutations carried in vectors pKAS32 and pKAS-Kan from *E*. *coli* donors were introduced into the *V*. *cholerae* genome by conjugation on LB plates. Transconjugants were selected for by plating on LB/Pb/Amp or Kan plates. Subsequent recombinants were selected on LB/Sm (5000 μg/ml) plates, followed by single colony isolation on LB/Sm (5000 μg/ml) plates [79]. Mutant strains carrying the desired mutations were screened and confirmed by PCR. All mutant strains were confirmed by sequencing at the Tufts University Core Facility.

### Bioluminescence assays

Bioluminescence assays were performed as described previously [12] with slight differences between the manual and automated assays. For manual assays, single colonies were grown overnight at 30 °C in LB containing appropriate antibiotics and the overnight cultures were diluted at least 100-fold in the same medium. Diluted cultures were grown at 30 °C and OD_600_ (1 ml of culture) and light production (0.2 ml of culture) were measured every 45–60 min until OD_600_ reached ~2.0 using a Thermo Scientific Evolution 201 UV-Visible Spectrophotometer and a BioTek Synergy HT Plate Reader, respectively. For automated assays, single colonies were grown in 200 μL of LB medium containing appropriate antibiotics in 96- or 384-well microplates for 20 hr at 30°C with aeration. OD_600_ and light production were measured every 30 min for at least 10 hr using a BioTek Synergy HT Plate Reader. Light production per cell was calculated from dividing light production by OD_600_.

### Random mutagenesis and screening of constitutively active CqsR mutants

GeneMorph II EZClone Mutagenesis kit (Agilent Technologies) was used for random mutagenesis; all steps were performed per the manufacturer’s instructions. The region encoding the predicted periplasmic ligand binding sensing domain (amino acids 31–297) was targeted for random mutagenesis. pEVS143 vector harboring the *cqsR* gene [12, 81] was isolated and mutagenized randomly used two PCR reactions. First, the LBD of *cqsR* was amplified with primers WNTP0548 (GAGTTATTGGGGGCTTGAAGT) and WNTP0549 (AATCCTTTTTCGATGTTGATAATTAAGTCG), using error-prone Mutazyme II DNA polymerase. For the second PCR, the original pEVS143-*cqsR* vector was mixed with the mutagenized inserts in excess, denatured and amplified with high-fidelity Phusion polymerase. This mixture was treated with Dpn I and transformed into competent XL10-Gold *E*. *coli* cells, and plated out entirely on LB/Kan plates to select for transformants. Transformants were pooled into separate libraries and a portion was conjugated into a quadruple QS receptor strain (Δ4, Δ*cqsS*, Δ*luxQ*, Δ*cqsR*, Δ*vpsS*) carrying a HapR-dependent bioluminescence reporter pBB1 [12] and pooled.

For screening of constitutively active CqsR mutant from the library, a portion of the Δ4/pBB1/pEVS143-*cqsR* mutant library was plated onto LB/Pb/Kan/Tet2 plates. Colonies were picked and transferred into 384-well clear bottom black microplates containing 50 μl of M9/Kan/Tet2/IPTG medium with 1% tryptone using QPix colony picking robot and grown at 30°C overnight. Approximately 5000 colonies were screened per round. OD_600_ and luminescence were measured with a BioTek Synergy HT Plate Reader. Mutants of interest that displayed low relative bioluminescence were selected and grown for 16 hr at 30°C with aeration. Single OD_600_ and bioluminescence measurements were collected again and compared to that of a strain expressing WT CqsR. The changes in the plasmid-borne *cqsR* of these mutants were determined by sequencing. To introduce specific amino acid changes, site directed mutagenesis on the plasmid-borne *cqsR* was performed using the QuikChange^™^ XL Site-Directed Mutagenesis Kit (Agilent) per manufacturer’s instructions.

### NMR metabolomics

Extracellular metabolite analysis of *V*. *cholerae* culture supernatants was performed as described previously [8]. Briefly, cultures of *V*. *cholerae* Δ3 *cqsR*^*+*^ (Δl*uxQ*, Δ*cqsS*, Δ*vpsS*) and *V*. *cholerae* strain Δ3 *cqsR*^*+*^ Δ*vca0136*, Δ*vc1554* (Δl*uxQ*, Δ*cqsS*, Δ*vpsS*, Δ*vca0136*, Δ*vc1554*) were grown in overnight in LB medium or M9 + 1% casamino acids and centrifuged at 10,000 rcf for 15 min. Supernatants were removed from the pellets, sterile filtered through a syringe filter (0.2 μM PES) and stored at −20°C until NMR analysis. Approximately five hundred microliters of the supernatants were transferred to 7-in., 600-MHz NMR tubes (Wilmad) and a final concentration of 0.05 mM 4,4-dimethyl-4-silapentane-1-sulfonic acid (DSS) in D2O was added to every sample as an internal standard. Plain LB medium was used as reference standard. An ^1^H NMR spectrum of each sample was collected at 25°C on a Bruker Avance 600 spectrometer by using 64 scans and a NOE1D pulse sequence. Data were processed and analyzed by using CHENOMX (version 8.0) for quantification of metabolites present in each sample. The average value and the standard error of the mean (SEM) were determined for at least three replicates.

### CqsR LBD purification

Full length CqsR as well as the region encoding the periplasmic region of CqsR (CqsR-LBD, residues 35–274) were PCR amplified with primer pairs WNTP0207 (ATATACCATGGCTATTCGCTCCTCGCTTAAAAAG) / WNTP0209 (CTCGAATTCGACTCTACCGATGGTAAAGATGGTTC) and WNTP0694 (GCCTGGTGCCGCGCGGCAGCGAAGTCCCATTTAGAAAAGAG) / WNTP695 (GCTTTGTTAGCAGCCGGATCTTAGATGTTGATAATTAAGTCGAAG) respectively from *V*. *cholerae* genomic DNA, while the vector pET28B was amplified with primers WNTP692 (GCTGCCGCGCGGCACCAG) and WNTP693 (GATCCGGCTGCTAACAAAG). Each insert was ligated into the plasmid backbone using Gibson assembly (NEB) and the resulting plasmids was sequenced and transformed into *E*. *coli* BL21(DE3) strains WN3657 andWN5327 respectively for protein overexpression and purification. MBP-fusions of WT CqsR-LBD and CqsR^D171V^-LBD were constructed by amplifying the LBD fragments from gDNA obtained from *Vibrio cholerae* strains WN3176 and WN5887 respectively using primers WNTP1290 (GGGATCGAGGGAAGGATTTCAGAATTCGAAGTCCCATTTAGAAAAGAGCTGAAAAA) and WNTP1291 (AGCTTGCCTGCAGGTCGACTCTAGATTAGATGTTGATAATTAAGTCGAAGATGATTTCATCGACA) and ligated into EcoRI and XbaI digested pMAL-c2X vector using Gibson assembly. The resulting plasmids were transformed in *E*. *coli* S17 λpir strains WN6173 and WN6180 respectively and verified by sequencing.

For CqsR-LBD overexpression, strain WN5327 was grown to OD_600_ ~ 0.5 and CqsR production was induced with 1 mM IPTG at 16 °C overnight. Cells after IPTG induction were collected by centrifugation and resuspended in binding buffer (50mM sodium phosphate buffer, pH 7.0; 300mM sodium chloride; 10mM imidazole, pH 7.7; 5% glycerol). Resuspended cells were then lysed with a fluidizer. Insoluble materials were removed by centrifugation (10000g, 4 °C, 1 hour) and subsequent filtering through a 0.45 μm filter. Cleared lysate was loaded onto His-Trap column (1 mL) equilibrated with binding buffer. The column was then washed with 20 mL binding buffer. Proteins were eluted with binding buffer containing 120 to 300 mM imidazole. Fractions containing CqsR-LBD were pooled and used in the *in vitro* biochemical assays described below.

For purification of the MBP fusion proteins, *E*. *coli* strain WN6173 (WT) or WN6180 (D171V) was grown in LB+0.2% glucose and MBP-CqsR expression was induced by addition of 0.3 mM IPTG for 3 hours at 30 °C. Cell pellet was resuspended in 20 mL ice-cold column buffer (20 mM Tris–HCl, pH 8.0, 200 mM NaCl, 1 mM EDTA, 5% glycerol) and disrupted by fluidizing. Insoluble proteins and cell debris were removed by centrifugation at 10,000 g for 15 min at 4 °C, and supernatants containing the target protein was collected. After filtration through a 0.45 μm filter, the supernatant was loaded into an amylose resin affinity column (NEB, MA, USA) equilibrated with column buffer. After loading, the column was washed with column buffer, then eluted with column buffer containing 10 mM maltose and 1 mL fractions were collected. Fractions were analyzed by SDS-PAGE for protein purity and then stored at − 20 °C for later use.

### Differential scanning fluorimetry

Differential scanning fluorimetry assays were performed using a BioRad CFX Connect Real‐Time PCR instrument [82]. For chemical screening, ligands were prepared by dissolving the contents of Biolog plates PM 1–4 in 50 μl of water to obtain a final concentration of around 10–20 mM. Each 20 μl standard assay contained 10 μM CqsR-LBD and SYPRO Orange at 5× concentration in a buffer containing Phosphate buffered saline pH 7.5 and 10% glycerol v/v. Two μl of the resuspended Biolog compounds were added to each well. Samples were heat denatured from 20°C to 90°C at a ramp rate of 1°C min^−1^. The protein unfolding curves were monitored by detecting changes in SYPRO Orange fluorescence. The first derivative values (−dF/dt) from the raw fluorescence data were used to determine the melting temperature (Tm). Each ligand was prepared as a 10× stock and 2 μl were added to each well and the experiments were conducted as described above. All experiments were performed in triplicate.

### MicroScale Thermophoresis

MicroScale Thermophoresis (MST) was performed using purified CqsR-LBD. For MST experiments, nickel affinity resin-purified CqsR-LBD was further purified using anion exchange chromatography (Source15Q—GE Healthcare) and size exclusion chromatography (S200 –GE Healthcare). Purified CqsR-LBD was concentrated to 4.3 mg/ml and stored in 50 mM sodium phosphate, pH 7.7, and 300 mM sodium chloride. CqsR-LBD was diluted to 20 μM and labeled using the Monolith NT Protein Labeling Ki RED-NHS (NanoTemper Technologies). Different concentrations of ethanolamine were incubated with 20 nM working stock solutions of labeled protein in the dark for 30 min at 4° C. After incubation, the samples were transferred into standard treated capillaries (NanoTemper Technologies) and read in a Monolith NT.115 Blue/Red instrument at room temperature using 20% LED and medium MST power. Binding affinities were calculated from three experiments.

### Chemical synthesis of EA metabolites

#### General experimental

Unless otherwise noted, all reactions were performed in flame-dried glassware under an atmosphere of nitrogen using dried reagents and solvents. All chemicals were purchased from commercial vendors and used without further purification. Anhydrous solvents were purchased from commercial vendors. Flash chromatography was performed using standard grade silica gel 60 230–400 mesh from SORBENT Technologies or was performed using a Biotage Flash Purification system equipped with Biotage silica gel or C18 columns. Analytical thin-layer chromatography was carried out using Silica G TLC plates, 200 μm with UV_254_ fluorescent indicator (SORBENT Technologies), and visualization was performed by staining and/or by absorbance of UV light. NMR spectra were recorded using a Varian Mercury Plus spectrometer (400 MHz for ^1^H-NMR; 100 MHz for ^13^C-NMR). Chemical shifts are reported in parts per million (ppm) and were calibrated according to residual protonated solvent. Mass spectroscopy data was collected using an Agilent 1100-Series LC/MSD Trap LC-MS or a Micromass Quattromicro with a Waters 2795 Separations Module LC-MS with acetonitrile containing 0.1% formic acid as the mobile phase in positive ionization mode. All final compounds were evaluated to be of greater than 90% purity by analysis of ^1^H-NMR and ^13^C-NMR unless otherwise indicated.

#### 2-((2-Hydroxyethyl)amino)acetic acid, HEGly

This compound was synthesized following the reported procedure [83] with modifications. Briefly, to a solution of ethanolamine (3.15 mL, 52.2 mmol) in anhydrous THF (53mL) in a oven-dried flask was added *i-*Pr_2_NEt (9.4 mL, 54.0 mmol). The resulting solution was cooled to 0°C and was treated with ethyl bromoacetate (5mL, 45.2mmol) dropwise. The mixture was stirred overnight with warming to room temperature and was concentrated *in vacuo* to remove solvent and was purified by silica gel flash chromatography to provide the ester, ethyl (2-hydroxyethyl)glycinate (3.5 g, 23.8 mmol, 53% yield). The ester, ethyl (2-hydroxyethyl)glycinate (3.5g, 23.8 mmol) was dissolved in a solution of acetone (119mL) and water (119mL) and was treated with LiOH (4.99g, 119.01mmol) and stirred at room temperature overnight. The reaction was quenched by the addition of 119mL of 1M HCl and was concentrated to dryness *in vacuo*. The residue was purified by slow recrystallization from H_2_O/EtOH at 5°C to provide 2-((2-Hydroxyethyl)amino)acetic Acid, HEGly (1.10g, 9.2mmol, 39% yield). Spectral data was consistent with previously reported data [83].

#### *N*-(2-hydroxyethyl)-2-((2-hydroxyethyl)amino)acetamide, HEHEAA

2-((2-Hydroxyethyl)amino)acetic Acid (HEGly, 87 mg, 0.73 mmol) was dissolved in H_2_O (200 uL) with warming. The solution was diluted with ACN (1.5 mL) and was treated sequentially with *i*-Pr_2_Net (250 uL, 1.46 mmol), 2-aminoethanol (88 uL, 1.46 mmol), 1-Hydroxybenzotriazole (HOBt, 197 mg, 1.46 mmol) and N-(3-Dimethylaminopropyl)-N’-ethylcarbodiimide hydrochloride (EDC, 279 mg, 1.46 mmol). The resulting mixture was heated to 55 °C and was stirred overnight. The reaction mixture was concentrated to dryness and purified using C18 flash chromatography to provide *N*-(2-hydroxyethyl)-2-((2-hydroxyethyl)amino)acetamide, HEHEAA. The product HEHEAA co-eluted with 1-(3-(dimethylamino)propyl)-3-ethylurea and was characterized and used in biological studies as an inseparable ca. 1:1 mixture based on ^1^H-NMR and ^13^C-NMR.

### Phos-tag gel electrophoresis and western blotting

*E*. *coli* BL21(DE3) strain WN3657 carrying pET28B vector containing full-length C-terminal 6x His-tagged CqsR protein, was grown till O.D. _600_ of ~0.5 in LB medium containing kanamycin at 30°C followed by induction with 1 mM IPTG and grown for a further 4 hours with and without 10mM ethanolamine before harvesting. Harvested cells were pelleted by centrifugation and resuspended in equal volumes of 20% glycerol before lysis at 37°C for 15 minutes in Laemmli sample buffer. Cell-lysates were run in parallel on a regular 8% SDS-PAGE gel as well as 8% SDS-PAGE gels containing 10μM MnCl2 and 50μM Phos-tag (Wako Pure Chemical Industries Ltd). Following electrophoresis, Phos-tag-acrylamide gels were soaked in wet transfer buffer (48mM Tris-base, 39mM glycine 20% methanol) containing 1mM EDTA for 15 minutes. For immunodetection, proteins were transferred to a nitrocellulose membrane (Biorad) by wet transfer. Membranes were blocked (5% non-fat milk powder, 0.1% Tween 20 in PBS) overnight and incubated with 6x His-Tag monoclonal (Invitrogen) primary antibodies for two hours. Membranes were then incubated with Donkey anti-Mouse IgG (H+L) cross-adsorbed secondary antibody, DyLight 800 (Invitrogen) for two hours before imaging using Odyssey Clx instrument (LiCor). Image processing and quantification was performed using Image Studio v5.2 (LiCor). To account for variation in protein loading across samples, band intensities observed in Phos-tag immunoblots were normalized using the respective band intensities from the same sample obtained from regular immunoblots.

### Ethics statement

All animal experiments were performed at and in accordance with the rules of the Tufts Comparative Medicine Services. All animal experiments were performed following the guidelines of the American Veterinary Medical Association (AVMA) as well as the Guide for the Care and Use of Laboratory Animals of the National Institutes of Health. All procedures were performed with approval of the Tufts University Institutional Animal Care and Use Committee (IACUC, Protocol# B2018-99). Euthanasia was performed in accordance with guidelines provided by the AVMA and was approved by the Tufts University IACUC.

### Infant mouse colonization model

*V*. *cholerae* bacterial cultures were grown aerobically for 16 hr in LB/Sm at 30°C. Strains were fed orally to 3- to 5-day-old CD-1 mice (Charles River Laboratories) at a concentration of 10^6^ colony forming units (CFU) in 50μL of LB. Prior to infection, infant mice were housed with ample food and water for at least 24 hr and monitored. For competition experiments, mutant strains were mixed equally with a wild-type Δ*lacZ* strain and serial dilutions of intestinal homogenate were plated on LB/Sm/X-Gal plates. Small intestine refers to the region of the gastrointestinal (GI) tract after the pylorus and before the caecum, while large intestine refers to the region that contains the caecum and colon. Competitive index (CI) was calculated as the ratio of output to input of the mutant strain relative to the wild-type. A minimum of eight infected animals were used to calculate CI. For ethanolamine dosing experiments, mice were infected with *V*. *cholerae* strains at time 0 and subsequently gavaged with 50μL 10mM ethanolamine or vehicle (LB) at 2 and 4 hours post-infection. Infected infant mice were then sacrificed 8 hours post bacterial inoculation and intestines were harvested and homogenized. *V*. *cholerae* colonization in the intestine was measured by plating serial dilutions of homogenate on LB/Sm plates and enumerating bacterial colonies the next day. *V*. *cholerae* colonization of the small and large intestine is presented as a single data point per mouse and data are graphed with the median and a 95% confidence interval. If the mutant strains were below the level of detection, it was assumed that there was 1 mutant CFU present at the next lowest dilution of the wild-type sample (indicated by open symbols in the figures). Two-tailed unpaired Student’s t tests assuming unequal variances were used for statistical analyses *P<0.05;**P<0.01;***P<0.001.

### Determining ethanolamine level in mouse small and large intestines

Infant mice (3–5 day old) were sacrificed and the small and large intestines were dissected. Each sample was longitudinally cut to expose the inner luminal surface, and contents were scraped and resuspended into 250 μL 1X phosphate buffered saline (pH 7.5). Ethanolamine present in each sample was derivatized and analyzed as described previously with modifications [43] [65]. A 100 μL of each sample was treated with 30 μL of 0.5 M NaHCO_3_, 200 μL of fresh dansyl chloride [20 mg/mL in acetone], and 20 μL of 1 M NaOH. The samples were incubated at ambient temperature for 20 minutes in the dark. Then 20 μL of 25% NH_4_OH was added and the volume was adjusted to 500 μL with acetonitrile. The sample was centrifuged at 11,000 rpm for 1 minute and the supernatant was used for further analysis. LC-MS/MS analysis of the dansyl monoethanolamine was performed using a Waters 2795 Separation Module coupled to a Micromass Quattro API *micro* MS. The tray was at ambient temperature. For each sample, 20 uL of derivatized product prepared as above was injected into a Phenomenex Synergi Max-RP column [50 x 4.60 mm, 4 micron]. Mobile phases used were: A, 0.1% formic acid in deionized water and B, pure acetonitrile. Column temperature was ambient and flow rate was 0.5 mL/min. A gradient was run from 5% B to 95% B over 4 min, the gradient was held at 95% B for 4.5 min, returned to 5% B over 0.5 min and equilibrate at 5% B for 1 min (10 minute run time). The flow from the HPLC column was diverted to waste for the first 2 mins of each run to exclude salts. Acquisition was performed in positive mode. Collision energy was set to 25V. A general MS/MS experiment was conducted to determine the fragmentation pattern and to choose the appropriate daughter ions for monitoring. Multiple reaction monitoring scan spectra of parent ions were acquired from 292.5 to 297.5 at unit mass resolution with maximum injection time set to 200 ms in one micro-scan. The range of selected atomic mass units corresponds to the [M+H]^+^ parent ion of dansyl monoethanolamine (Mi 295). MRM transitions were found 295/280, 295/171, and 295/157. These transitions were used for dansyl monoethanolamine detection. The target was eluted at 4.87 min. The concentration of ethanolamine in each sample was inferred by the ion counts using a series of standards made from pure ethanolamine. To compare the relative amounts of ethanolamine present in the small and large intestines, we took into account the fact that the luminal surface area of the murine small intestine ranges from 9–20 times larger than that of the large intestine based on two independently published reports [39, 40].

### Quantification of *tcpA* expression in the large intestine

RNA was isolated at 8hr post-infection from large intestine samples of mice infected with WT *V*. *cholerae*, Δ3 *cqsR*^*+*^, Δ3 *cqsR*^*D171V*^ (n = 4 per group). Large intestine samples were homogenized in Trizol (1ml) and purified using the Direct-zol^®^ RNA mini-prep kit (Zymo) following the manufacturer’s instructions. DNA was removed using a Turbo DNA-*free*^™^ kit (Thermo Fisher). cDNA was synthesized from 2μg RNA using 2pmol of gene-specific primers using SuperScript First Strand Synthesis System for qPCR (Invitrogen). Controls lacking reverse transcriptase were included. qRT-PCR experiments were performed using SYBR Select Master Mix (Invitrogen). Each reaction contained 250nM primers, 10ng template. For each sample, the mean cycle threshold of the test transcript was normalized to that of 16S RNA. The primer sequences for *tcpA* (CTACCGCAAACGCAAATGCT / GGTCAAGCCACCGACTGTAA) and 16S RNA (CGTAAAGCGCATGCAGGTG / CTTCGCCACCGGTATTCCTT) used in this study were taken from previously published reports [84] [85].

## Supporting information

S1 Table List of strains used in this study. (DOCX)

S2 Table Binding activity of CqsR to common byproducts of ethanolamine. (DOCX)

S3 Table Structures of compounds tested for CqsR binding with differential scanning fluorimetry. (DOCX)

S1 Fig Large intestine colonization is dependent on TcpA and LuxO activation. Competitive indexes (CI) were determined between wild-type ΔlacZ and the indicated *V. cholerae* mutants per large intestine homogenate collected from infant mice 24 hr post-infection (n = 8). Each symbol represents the CI in an individual mouse and data is represented with horizontal lines indicating the median for each group. Open symbols represent data below the limit of detection for the mutant strain. In that case, it was assumed that there was one mutant CFU present at the next lowest dilution to calculate the CIs.
(TIF)

S2 Fig Effect of ethanolamine and its analogs on qrr4 transcription. Normalized bioluminescence production (lux/OD_600_) using a P*_qrr_*_4_-*lux* reporter was measured in Δ3 cqsR^+^ strain in the presence of 10 mM ethanolamine, L-alaninol, D-alaninol, or serinol. Blank indicates LB medium without added compound. Each figure shows a representative profile of each condition with three biological replicates. Each experiment was performed independently at least two times.
(TIF)

S3 Fig Ethanolamine induces high cell density QS response in a CqsR-specific manner. HapR-dependent bioluminescence profiles (lux/OD_600_) were measured in A) Δ3 luxQ^+^, B) Δ3 cqsS^+^, C) Δ3 cqsR^+^, and D) Δ3 vpsS^+^, in LB medium and LB medium containing 10 mM ethanolamine. Each figure shows a representative profile of each condition with two biological replicates. Each experiment was performed independently at least two times.
(TIF)

S4 Fig Factors affecting dynamics of intestinal colonization. CFU counts were enumerated by counting white colonies representing WT *V. cholerae* (ΔlacZ) and blue colonies for A) Δ3 cqsR^+^ or B) Δ3 cqsR^D171V^ obtained from plating of small intestinal (SI) and large intestinal (LI) homogenates of infant mice 24 hr post infection (n = 8) on selective plates containing Sm and X-gal. These cfu counts were used to calculate competitive indices shown in Fig 6C of the main text. C) TcpA expression was enumerated by qRT-PCR in large intestinal homogenates collected from mice (n = 4) infected singly with WT *V. cholerae*, Δ3 cqsR^+^ or Δ3 cqsR^D171V^ mutant strains and normalized by 16s rRNA expression for each strain. Data represent mean fold change ± standard deviation relative to tcpA expression in the WT strain. D) Normalized ethanolamine levels from large intestinal and small intestinal contents of uninfected mice (n = 7) were enumerated by comparing LC-MS/MS ion counts of derivatized dansyl monoethanolamine obtained from each sample to a dansyl monoethanolamine reference standard. Data presented has been normalized to the relative luminal surface area of small or large intestine with a ratio of 9 or 20 as previously reported [39, 40].
(TIF)

S5 Fig Effect of ethanolamine on WT *Vibrio cholerae*. HapR-dependent bioluminescence profiles (lux/OD_600_) were measured in WT and Δ3 cqsR^+^ strains in LB medium and LB medium containing 10 mM ethanolamine in duplicate. Each experiment was performed independently at least two times.
(TIF)

S6 Fig P*_qrr_*_4_-*lux* reporter assays. Normalized bioluminescence production (lux/OD_600_) using a P_*qrr*__4_-*lux* reporter was measured in A) Δ3 cqsR^+^ or Δ3 cqsR ^+^ Δvc1554 Δvca0136 strains grown in LB medium in the absence or presence of 10mM ethanolamine B) Δ3 cqsR^+^ strains and C) Δ3 cqsR ^+^ Δvc1554 Δvca0136 strains grown in 20% 5x LB + 80% 1x LB medium (black lines) or 20% 5x LB + 80% 1x Δ3 cqsR^+^ spent culture medium (red lines) or 20% 5x LB + 80% 1x Δ3 cqsR^+^ Δvc1554 Δvca0136 spent culture medium (blue lines). Each experiment was performed independently at least two times.
(TIF)

S7 Fig Ethanolamine is not utilized by *Vibrio cholerae* as a sole carbon or nitrogen source. Growth of WT *Vibrio cholerae* was assessed in M9 medium containing A) NH_4_Cl (5g/L) or B) no NH_4_Cl as an inorganic nitrogen source in addition to 10 mM glucose, 10 mM ethanolamine or both. While growth was observed in the presence of glucose as a sole carbon source, ethanolamine alone did not enable such growth in the presence of NH_4_Cl as a nitrogen source. Similarly, when NH_4_Cl was absent from the medium, EA was not utilized as a nitrogen source, since no growth was observed in the presence of EA alone or EA and glucose combined. Each experiment was performed in triplicates independently at least two times.
(TIF)
