# Supplementary material for: Parallel quorum-sensing system in Vibrio cholerae prevents signal interference inside the host
Source: PLoS Pathog. 2020 Feb 14;16(2):e1008313. doi: 10.1371/journal.ppat.1008313 (PMC7046293; doi:10.1371/journal.ppat.1008313)
Supplement: S1 Table — (DOCX) [file ppat.1008313.s001.docx]

| **Strain Number** | **Organism** | **Genotype** | **Ab Resistance^a^** | **Source** |
| --- | --- | --- | --- | --- |
| **WN3176** | *V. cholerae* | Δ*cqsS* Δ*luxQ* Δ*vpsS* | Sm | [1] |
| **WN3208** | *V. cholerae* | Δ*cqsS* Δ*luxQ* Δ*vpsS* / pBB1 | Sm Tet | [1] |
| **WN3357** | *V. cholerae* | Δ*cqsS* Δ*luxQ* Δ*cqsR* /pBB1 | Sm Tet | [1] |
| **WN3649** | *V. cholerae* | Δ*cqsS* Δ*cqsR* Δ*vpsS* /pBB1 | Sm Tet | [1] |
| **WN3651** | *V. cholerae* | Δ*luxQ* Δ*cqsR* Δ*vpsS* /pBB1 | Sm Tet | [1] |
| **WN3354** | *V. cholerae* | Δ*cqsS* Δ*luxQ* Δ*vpsS* Δ*cqsR* | Sm | [1] |
| **WN4899** | *V. cholerae* | Δ*cqsS* Δ*luxQ* Δ*vpsS* Δ*cqsR* / pEVS143-*cqsR* / pBB1 | Sm Kan Tet | This study |
| **WN5267** | *V. cholerae* | Δ*cqsS* Δ*luxQ* Δ*vpsS* Δ*cqsR* / *pEVS143-cqsR*^R49S^  pBB1 | Sm Kan Tet | This study |
| **WN5275** | *V. cholerae* | Δ*cqsS* Δ*luxQ* Δ*vpsS* Δ*cqsR* / pEVS143-*cqsR*^D198V^ / pBB1 | Sm Kan Tet | This study |
| **WN5277** | *V. cholerae* | Δ*cqsS* Δ*luxQ* Δ*vpsS* Δ*cqsR* / pEVS143-*cqsR*^L217S^ / pBB1 | Sm Kan Tet | This study |
| **WN5279** | *V. cholerae* | Δ*cqsS* Δ*luxQ* Δ*vpsS* Δ*cqsR* / pEVS143-*cqsR*^V219E^ / pBB1 | Sm Kan Tet | This study |
| **WN5281** | *V. cholerae* | Δ*cqsS* Δ*luxQ* Δ*vpsS* Δ*cqsR* / pEVS143-*cqsR*^A259V^ / pBB1 | Sm Kan Tet | This study |
| **WN5285** | *V. cholerae* | Δ*cqsS* Δ*luxQ* Δ*vpsS* Δ*cqsR* / pEVS143-*cqsR*^H262Y^ / pBB1 | Sm Kan Tet | This study |
| **WN5318** | *V. cholerae* | Δ*cqsS* Δ*luxQ* Δ*vpsS* Δ*cqsR* / pEVS143-*cqsR*^F268I^ / pBB1 | Sm Kan Tet | This study |
| **WN5354** | *V. cholerae* | Δ*cqsS* Δ*luxQ* Δ*vpsS* Δ*cqsR* / pEVS143-*cqsR*^D171V^ / pBB1 | Sm Kan Tet | This study |
| **WN5781** | *V. cholerae* | Δ*cqsS* Δ*luxQ* Δ*vpsS* Δ*vc1554* Δ*vca0136* | Sm | This study |
| **WN5784** | *V. cholerae* | Δ*cqsS* Δ*luxQ* Δ*vpsS* Δ*vc1554* Δ*vca0136*/pBB1 | Sm Tet | This study |
| **WN5886** | *V. cholerae* | Δ*cqsS* Δ*luxQ* Δ*vpsS cqsR*^D198V^ | Sm | This study |
| **WN5887** | *V. cholerae* | Δ*cqsS* Δ*luxQ* Δ*vpsS cqsR*^D171V^ | Sm | This study |
| **WN5890** | *V. cholerae* | Δ*cqsS* Δ*luxQ* Δ*vpsS cqsR*^D198V^*/ pBB1* | Sm Tet | This study |
| **WN5891** | *V. cholerae* | Δ*cqsS* Δ*luxQ* Δ*vpsS cqsR*^D171V^*/ pBB1* | Sm Tet | This study |
| **WN5976** | *V. cholerae* | Δ*cqsS* Δ*luxQ* Δ*vpsS /pBK1003 (Pqrr4-lux)* | Sm Cm | This study |
| **WN5979** | *V. cholerae* | Δ*cqsS* Δ*luxQ* Δ*vpsS Δvc1554 Δvca0136 / pBK1003* | Sm Cm | This study |
| **WN5980** | *V. cholerae* | Δ*cqsS* Δ*luxQ* Δ*vpsS cqsR*^D198V^ */ pBK1003* | Sm Cm | This study |
| **WN5981** | *V. cholerae* | Δ*cqsS* Δ*luxQ* Δ*vpsS cqsR*^D171V^ */ pBK1003* | Sm Cm | This study |
| **WN048** | *E. coli* | S17-1 *λpir* / pBB1 | Tet | [1] |
| **WN3657** | *E. coli* | BL21(DE3) / pET28b *cqsR* | Kan | This study |
| **WN3705** | *E. coli* | S17-1 λ*pir* / pEVS143-*cqsR* | Kan | [1] |
| **WN5233** | *E. coli* | XL10-Gold / *pEVS143-cqsR*^V219E^ | Kan | This study |
| **WN5236** | *E. coli* | XL10-Gold / *pEVS143-cqsR*^A259V^ | Kan | This study |
| **WN5239** | *E. coli* | XL10-Gold / *pEVS143-cqsR*^H262Y^ | Kan | This study |
| **WN5242** | *E. coli* | XL10-Gold */ pEVS143-cqsR*^R49S^ | Kan | This study |
| **WN5248** | *E. coli* | XL10-Gold / *pEVS143-cqsR*^D198V^ | Kan | This study |
| **WN5250** | *E. coli* | XL10-Gold / *pEVS143-cqsR*^L217S^ | Kan | This study |
| **WN5318** | *E. coli* | XL10-Gold / *pEVS143-cqsR*^F268I^ | Kan | This study |
| **WN5326** | *E. coli* | BL21DE3/ pET28b *cqsR* -LBD | Kan | This study |
| **WN5344** | *E. coli* | XL10-Gold */ pEVS143-cqsR*^D171V^ | Kan | This study |
| **WN5745** | *E. coli* | S17 λpir DAP- / pKAS::Kan *Δvc1554* | Kan | This study |
| **WN5746** | *E. coli* | S17 λpir DAP- / pKAS::Kan *Δvca0136* | Kan | This study |
| **WN5880** | *E. coli* | S17 λpir DAP- / pKAS::Kan *cqsR*^D198V^ | Kan | This study |
| **WN5881** | *E. coli* | S17 λpir DAP- / pKAS::Kan *cqsR*^D171V^ | Kan | This study |
| **WN6173** | *E. coli* | S17 λpir /pMAL *cqsR*-LBD | Amp | This study |
| **WN6180** | *E. coli* | S17 λpir /pMAL *cqsR*^D171V^-LBD | Amp | This study |

^a^ Sm = Streptomycin, Kan = Kanamycin, Cm = Chloramphenicol, Tet = Tetracycline

**REFERENCES**

1. Jung SA, Chapman CA, Ng WL. Quadruple quorum-sensing inputs control Vibrio cholerae virulence and maintain system robustness. PLoS Pathog. 2015;11(4):e1004837. Epub 2015/04/16. doi: 10.1371/journal.ppat.1004837. PubMed PMID: 25874462; PubMed Central PMCID: PMCPMC4398556.
