# Supplementary material for: Parallel quorum-sensing system in Vibrio cholerae prevents signal interference inside the host
Source: PLoS Pathog. 2020 Feb 14;16(2):e1008313. doi: 10.1371/journal.ppat.1008313 (PMC7046293; doi:10.1371/journal.ppat.1008313)
Supplement: S2 Table — (DOCX) [file ppat.1008313.s002.docx]

| **Compound** | **Tm^a^** |
| --- | --- |
| None | 47.00 |
| N-(2-hydroxyethyl)-acetamide | 48.00 |
| N,N'-Bis(2-hydroxyethyl)-ethanediamide | 49.00 |
| N-(2-hydroxyethyl)-2-[(2-hydroxyethyl)amino]-acetamide | 47.67 [0.57] |
| N-(2-hydroxyethyl)ethylenediamine | 47.67 [0.57] |
| N-(2-hydroxyethyl) imidazolidone | 47.00 |
| N-(2-hydroxyethyl)-glycine | 47.33 [0.57] |
| N-(2-hydroxyethyl)-imidazole | 47.33 [0.57] |
| N-(2-hydroxyethyl)-formamide | 50.00 |

^a^Data shown as average of 3 replicates in the presence of 1 mM tested compounds. Square brackets indicate standard deviation where applicable.
