# Supplementary material for: Parallel quorum-sensing system in Vibrio cholerae prevents signal interference inside the host
Source: PLoS Pathog. 2020 Feb 14;16(2):e1008313. doi: 10.1371/journal.ppat.1008313 (PMC7046293; doi:10.1371/journal.ppat.1008313)
Supplement: S3 Table — (DOCX) [file ppat.1008313.s003.docx]

| **Compound** | **Structure^a^** |
| --- | --- |
| None | - |
| Ethanolamine [2-aminoethanol] |  |
| Cysteamine [2-aminoethanethiol] |  |
| 1-propanamine |  |
| β-alanine [2-Carboxyethylamine] |  |
| Mercaptoethanol [2-Hydroxyethanethiol] |  |
| 1-Propanol |  |
| 2-Methylaminoethanol |  |
| 2-Dimethylaminoethanol |  |
| Choline [(2-Hydroxyethyl)trimethyl ammonium] |  |
| (S)-(+)-1-Amino-2-propanol |  |
| (R)-(−)-1-Amino-2-propanol |  |
| L-alaninol [(S)-(+)-2-Amino-1-propanol] |  |
| D-alaninol [(R)-(−)-2-Amino-1-propanol] |  |
| Serinol [2-Amino-1,3-propanediol] |  |

^a^Functional groups displayed in red are the ones that vary from the base compound ethanolamine.
