# Supplementary figures and images for: Parallel quorum-sensing system in Vibrio cholerae prevents signal interference inside the host
Source: PLoS Pathog. 2020 Feb 14;16(2):e1008313. doi: 10.1371/journal.ppat.1008313 (PMC7046293; doi:10.1371/journal.ppat.1008313)

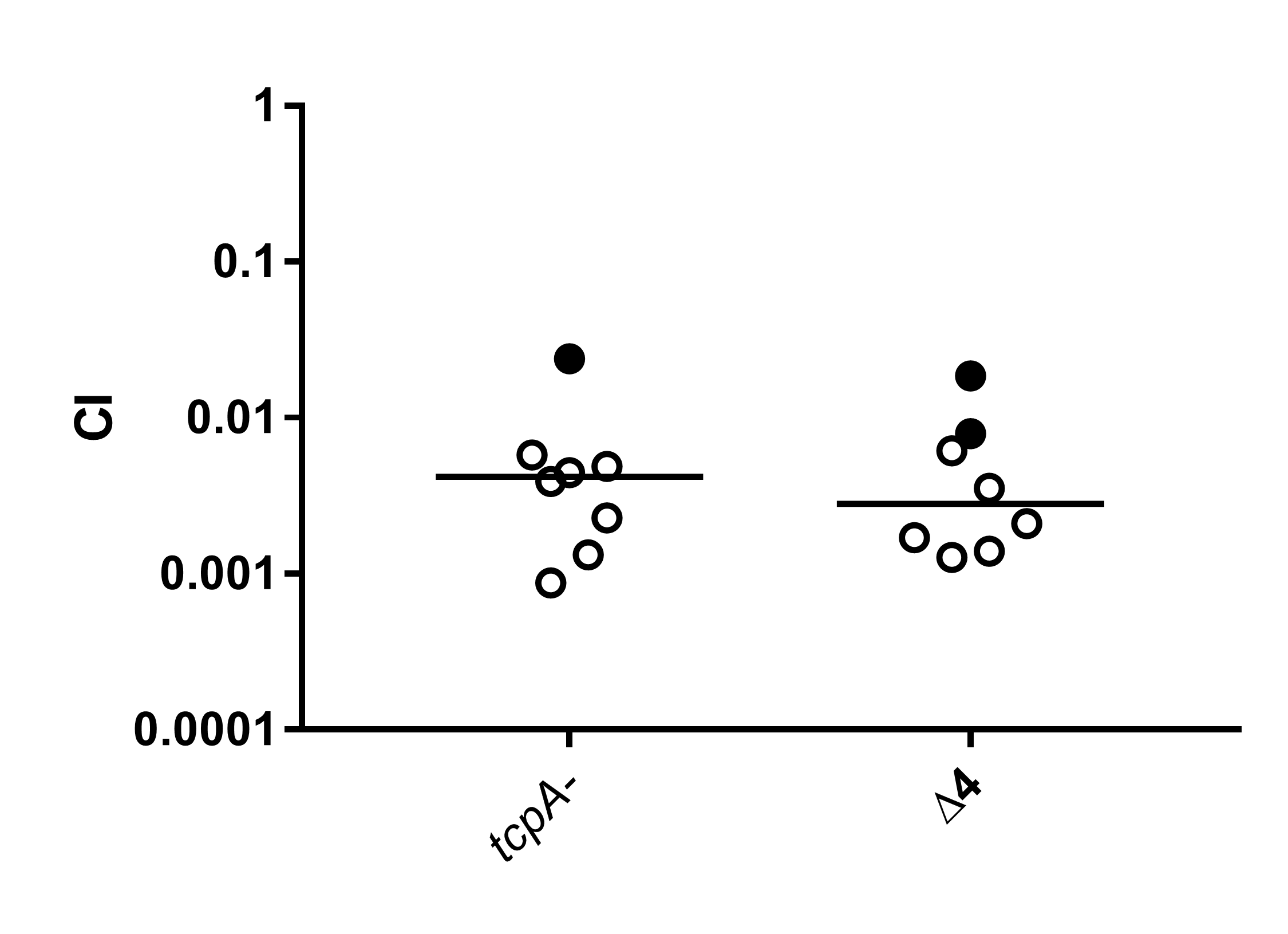

Supplement: S1 Fig — Competitive indexes (CI) were determined between wild-type ΔlacZ and the indicated V. cholerae mutants per large intestine homogenate collected from infant mice 24 hr post-infection (n = 8). Each symbol represents the CI in an individual mouse and data is represented with horizontal lines indicating the median for each group. Open symbols represent data below the limit of detection for the mutant strain. In that case, it was assumed that there was one mutant CFU present at the next lowest dilution to calculate the CIs. (TIF) [file ppat.1008313.s004.tif]

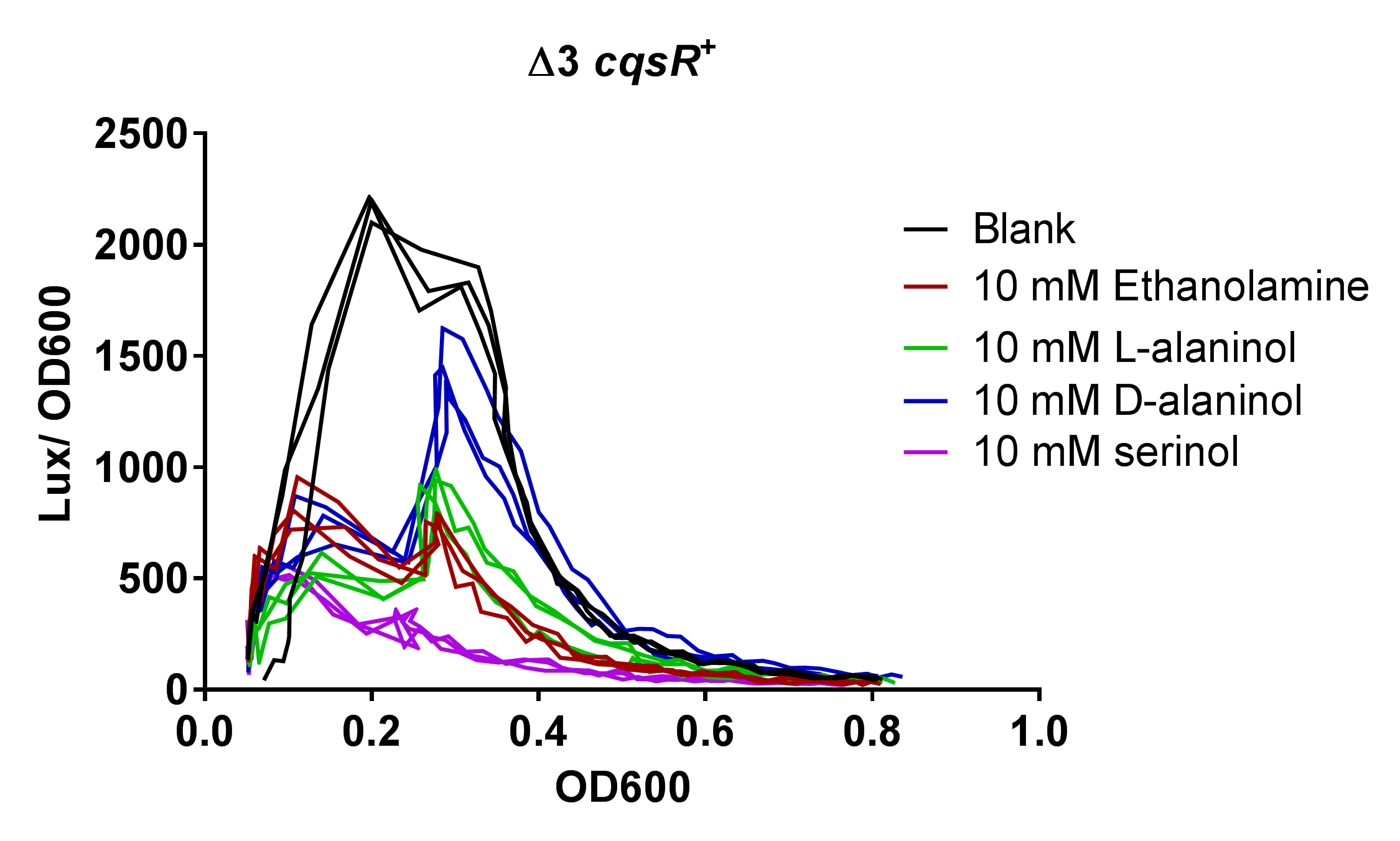

Supplement: S2 Fig — Normalized bioluminescence production (lux/OD600) using a Pqrr4-lux reporter was measured in Δ3 cqsR+ strain in the presence of 10 mM ethanolamine, L-alaninol, D-alaninol, or serinol. Blank indicates LB medium without added compound. Each figure shows a representative profile of each condition with three biological replicates. Each experiment was performed independently at least two times. (TIF) [file ppat.1008313.s005.tif]

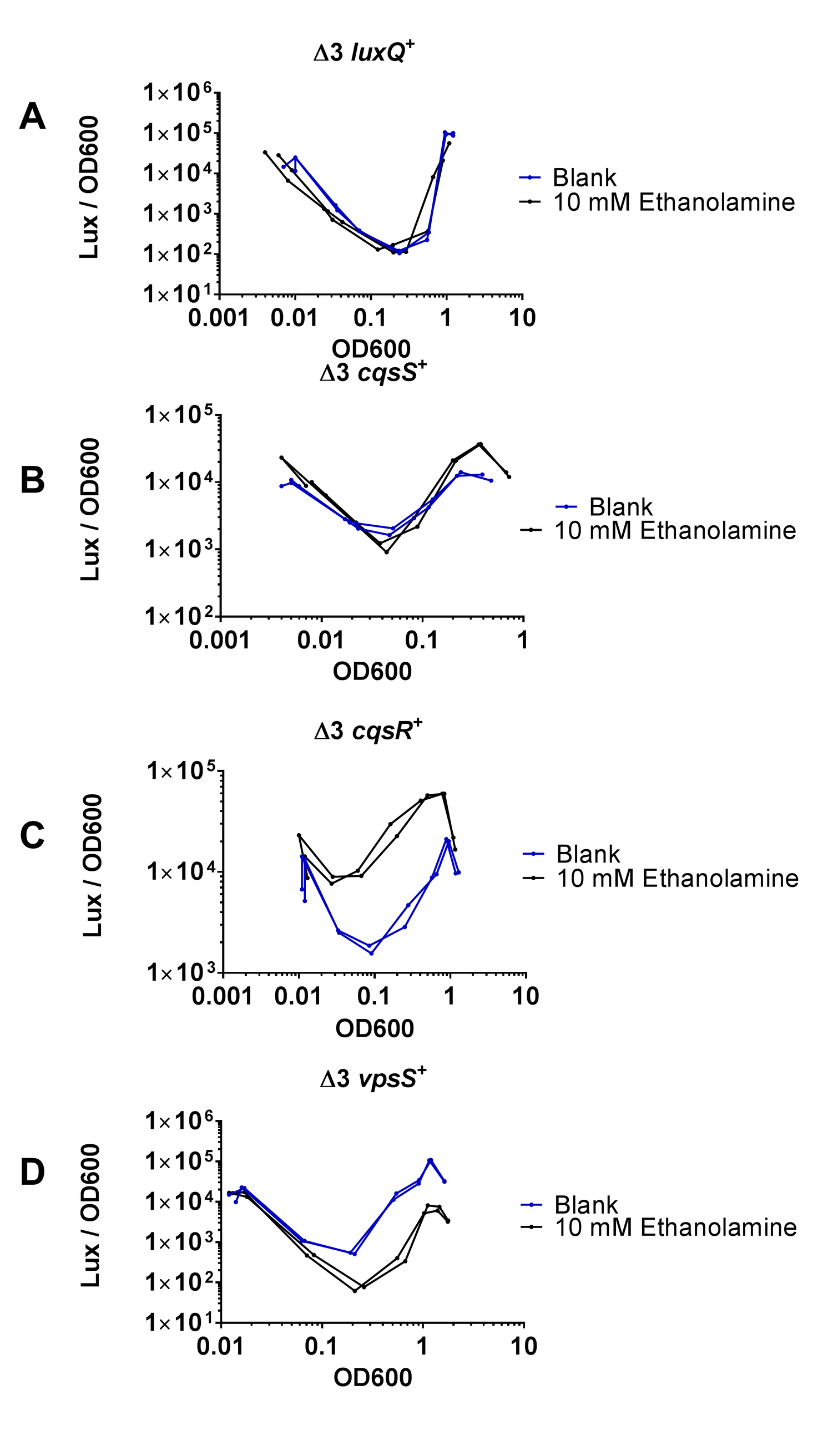

Supplement: S3 Fig — HapR-dependent bioluminescence profiles (lux/OD600) were measured in A) Δ3 luxQ+, B) Δ3 cqsS+, C) Δ3 cqsR+, and D) Δ3 vpsS+, in LB medium and LB medium containing 10 mM ethanolamine. Each figure shows a representative profile of each condition with two biological replicates. Each experiment was performed independently at least two times. (TIF) [file ppat.1008313.s006.tif]

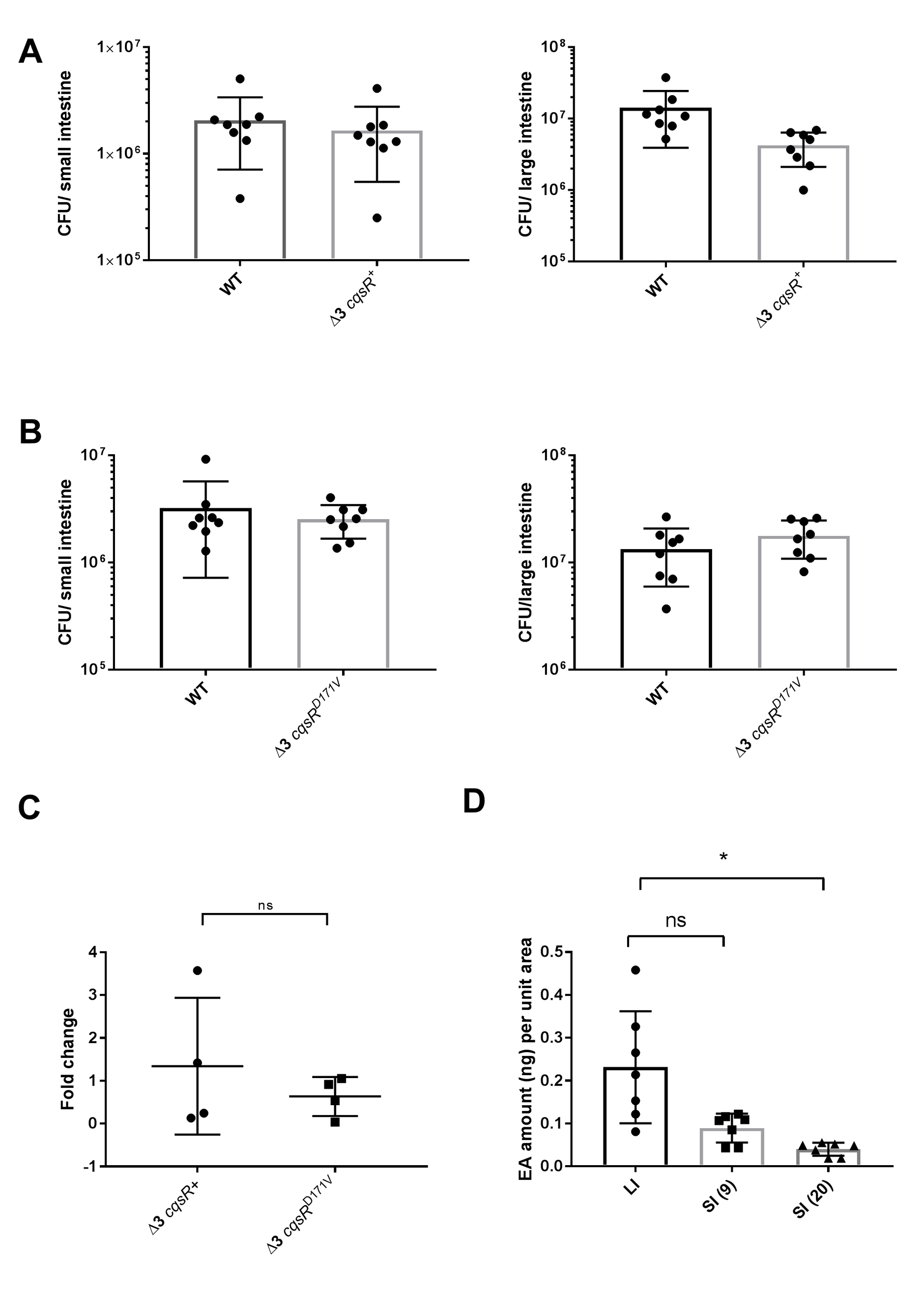

Supplement: S4 Fig — CFU counts were enumerated by counting white colonies representing WT V. cholerae (ΔlacZ) and blue colonies for A) Δ3 cqsR+ or B) Δ3 cqsRD171V obtained from plating of small intestinal (SI) and large intestinal (LI) homogenates of infant mice 24 hr post infection (n = 8) on selective plates containing Sm and X-gal. These cfu counts were used to calculate competitive indices shown in Fig 6C of the main text. C) TcpA expression was enumerated by qRT-PCR in large intestinal homogenates collected from mice (n = 4) infected singly with WT V. cholerae, Δ3 cqsR+ or Δ3 cqsRD171V mutant strains and normalized by 16s rRNA expression for each strain. Data represent mean fold change ± standard deviation relative to tcpA expression in the WT strain. D) Normalized ethanolamine levels from large intestinal and small intestinal contents of uninfected mice (n = 7) were enumerated by comparing LC-MS/MS ion counts of derivatized dansyl monoethanolamine obtained from each sample to a dansyl monoethanolamine reference standard. Data presented has been normalized to the relative luminal surface area of small or large intestine with a ratio of 9 or 20 as previously reported [39, 40]. (TIF) [file ppat.1008313.s007.tif]

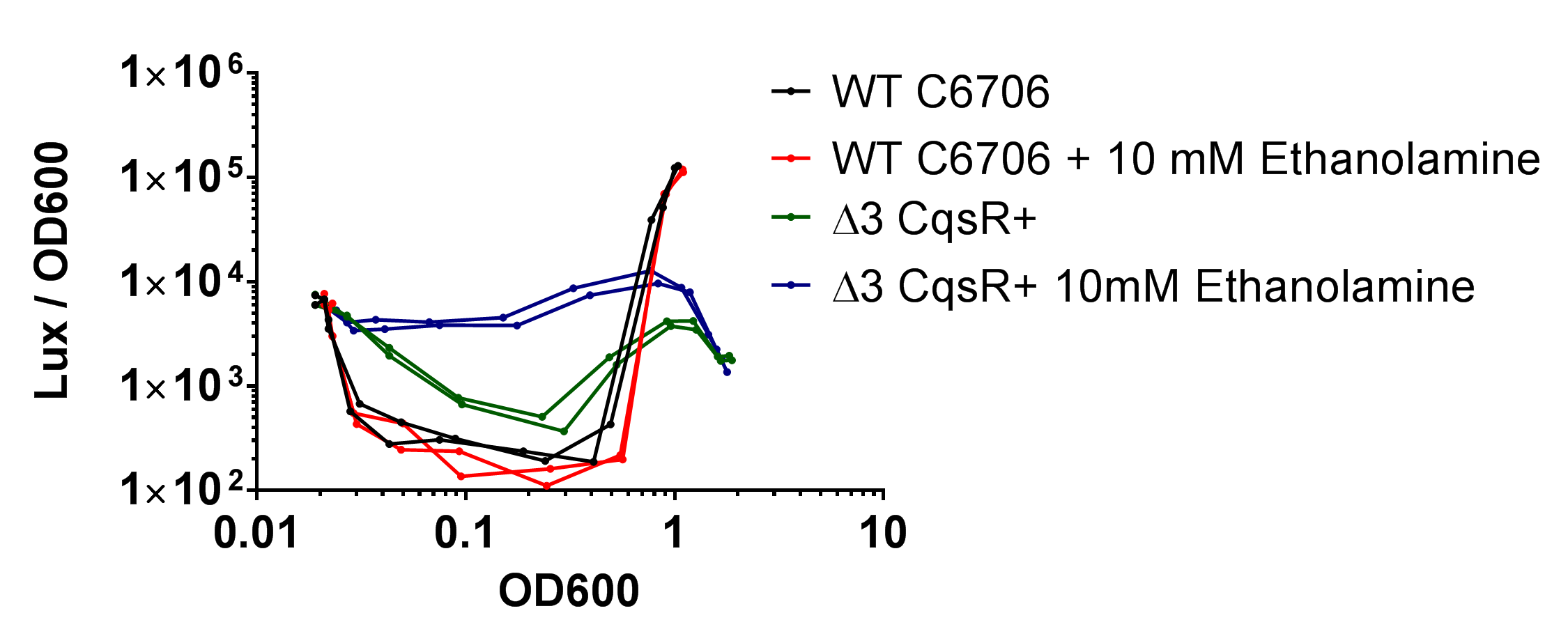

Supplement: S5 Fig — HapR-dependent bioluminescence profiles (lux/OD600) were measured in WT and Δ3 cqsR+ strains in LB medium and LB medium containing 10 mM ethanolamine in duplicate. Each experiment was performed independently at least two times. (TIF) [file ppat.1008313.s008.tif]

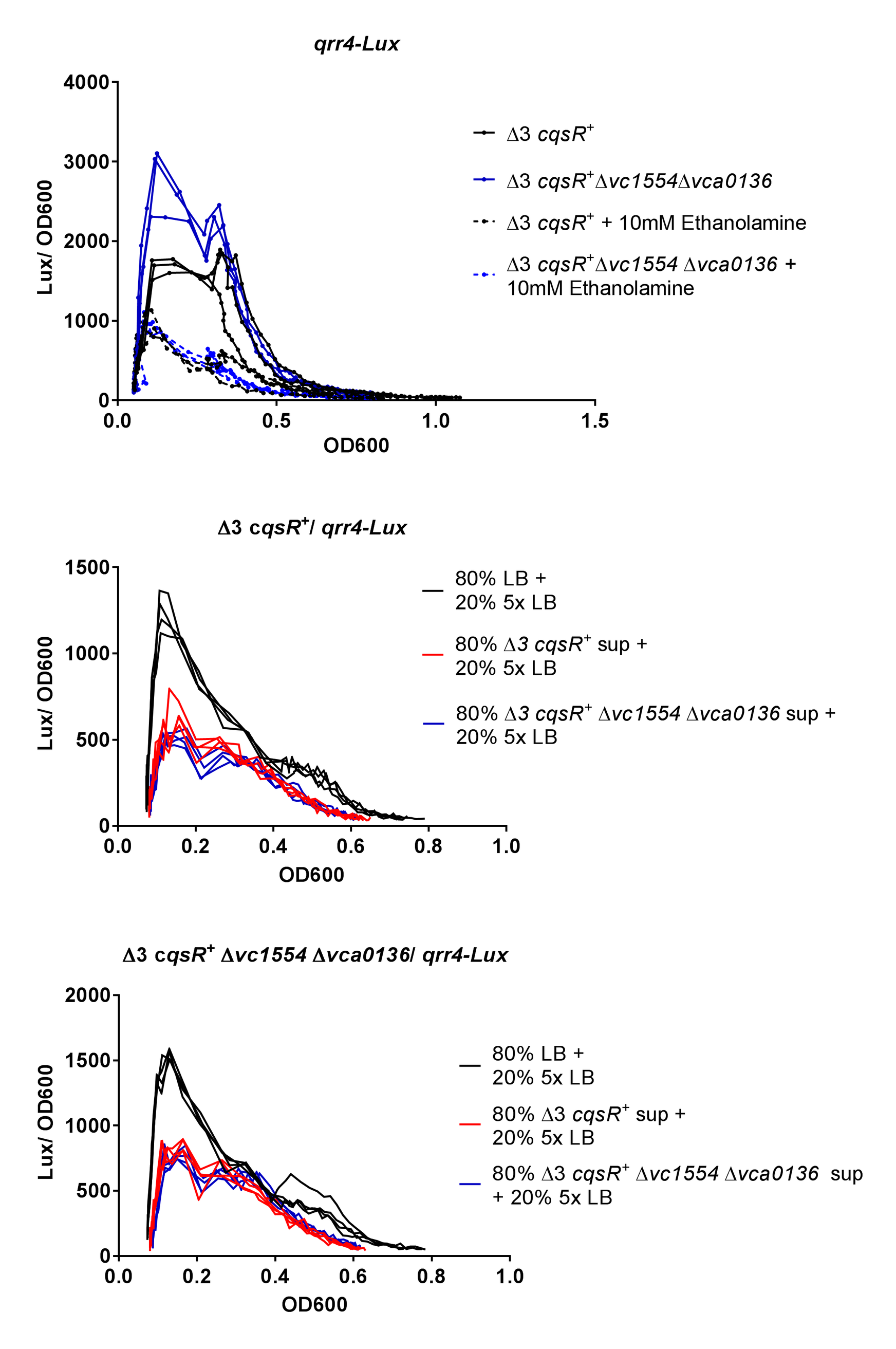

Supplement: S6 Fig — Normalized bioluminescence production (lux/OD600) using a Pqrr4-lux reporter was measured in A) Δ3 cqsR+ or Δ3 cqsR + Δvc1554 Δvca0136 strains grown in LB medium in the absence or presence of 10mM ethanolamine B) Δ3 cqsR+ strains and C) Δ3 cqsR + Δvc1554 Δvca0136 strains grown in 20% 5x LB + 80% 1x LB medium (black lines) or 20% 5x LB + 80% 1x Δ3 cqsR+ spent culture medium (red lines) or 20% 5x LB + 80% 1x Δ3 cqsR+ Δvc1554 Δvca0136 spent culture medium (blue lines). Each experiment was performed independently at least two times. (TIF) [file ppat.1008313.s009.tif]

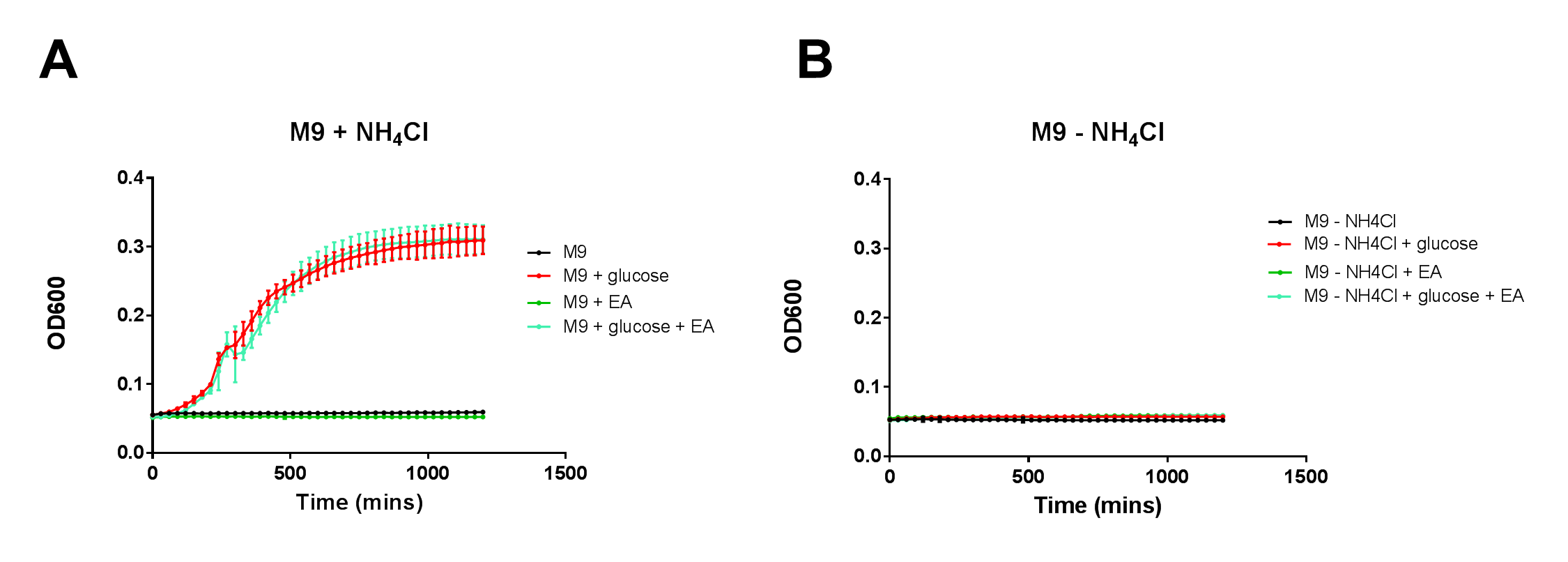

Supplement: S7 Fig — Growth of WT Vibrio cholerae was assessed in M9 medium containing A) NH4Cl (5g/L) or B) no NH4Cl as an inorganic nitrogen source in addition to 10 mM glucose, 10 mM ethanolamine or both. While growth was observed in the presence of glucose as a sole carbon source, ethanolamine alone did not enable such growth in the presence of NH4Cl as a nitrogen source. Similarly, when NH4Cl was absent from the medium, EA was not utilized as a nitrogen source, since no growth was observed in the presence of EA alone or EA and glucose combined. Each experiment was performed in triplicates independently at least two times. (TIF) [file ppat.1008313.s010.tif]
